# Unlocking the potential of *Pseudomonas aeruginosa* QS intermediates as antimicrobial synergists against three multidrug-resistant enteric bacteria

**DOI:** 10.3389/fmicb.2026.1726554

**Published:** 2026-03-05

**Authors:** Saba Kiran, Alaa S. Alhegaili, Noura Al-Dayan, Zubera Naseem, Waqar Siddique, Itra Ayoub, Shoaib Iqbal, Sobia Jabeen, Fazal-E Habib, Saman Taj, Ashfaq Hussain, Rizwan Bashir, Yasra Sarwar, Aamir Ali, Waqar Rauf, Georg Jander, Mazhar Iqbal

**Affiliations:** 1 Health Biotechnology Division, National Institute for Biotechnology and Genetic Engineering College, Pakistan Institute of Engineering and Applied Sciences (NIBGE-C, PIEAS), Faisalabad, Punjab, Pakistan; 2 Boyce Thompson Institute, Ithaca, NY, United States; 3 Department of Medical Laboratory, College of Applied Medical Sciences, Prince Sattam bin Abdulaziz University, Alkharj, Saudi Arabia

**Keywords:** anti-Enterobacteriaceae, antimicrobial resistance, antimicrobial synergy, *Pseudomonas aeruginosa*, secondary metabolites

## Abstract

**Introduction:**

Antimicrobial resistance in the Enterobacteriaceae poses a global health concern by jeopardizing the effectiveness of antibiotics. The scarcity of new antibiotics has prompted increased interest in natural bioactive secondary metabolites derived from microbial sources and their co-action with existing antimicrobials.

**Methods:**

In this study, we investigated the bioactivity of crude extracts from *Pseudomonas aeruginosa* MC9 (accession no. MK530186) and evaluated the *in-vitro* antimicrobial-augmenting efficacy of its quorum sensing (QS) effectors against multidrug-resistant strains of *Salmonella* Typhi (*S.* Typhi-29C), *Salmonella* Typhimurium (*S.* Typhimurium-W20), and *Escherichia coli* (*E. coli* SS1). Mass spectrometry was used to identify secondary metabolites, and combination assays followed by growth curve analysis were performed to assess interaction effects under sub-inhibitory conditions.

**Results:**

The MC9 extract exhibited inhibition zones of 26±1.5, 24±1, and 19±1.5 mm, with minimum inhibitory concentrations of 16, 32, and 256 mg/mL against S. Typhimurium-W20, S. Typhi-29C, and E. coli-E92, respectively. Mass spectrometric analysis revealed the presence of 5-methyl-1(5H)-phenazinone (pyocyanin), rhamnolipids, 4-hydroxy-2heptylquinoline (PQS), and 2-heptyl-3-hydroxy-4(1H)-quinolone (HHQ). Notably, pyocyanin and rhamnolipids exhibited significant antimicrobial activities across a concentration range from 0.04 mg/mL to 50 mg/mL, whereas HHQ and PQS showed no anti-Enterobacteriaceae activity up to 5 mg/mL. Combination assays demonstrated that all four QS effectors potentiate the activity of conventional antibiotics. Pyocyanin showed the highest synergistic effect, with a 300% increase in the inhibition zone when combined with sulfamethoxazole/trimethoprim (23.75/1.25 µg/mL) against *S.* Typhimurium-W20. Rhamnolipids exhibited a 106% increase in synergy with ceftriaxone (30 μg/mL) against *E. coli*-SS1, whereas HHQ (10 μg/mL) showed a 257% increase with ampicillin (10 μg/mL) against *E. coli*-SS1. PQS displayed the highest synergistic effect of 109% with amoxicillin clavulanic acid (30 μg/mL) against *E. coli*-SS1. Moreover, growth curve analysis revealed a dose-dependent reduction in bacterial growth with sub-inhibitory concentrations of antimicrobials, particularly for the combinations exhibiting the highest synergy across the QS effectors.

**Discussion:**

These findings demonstrate the potential of the QS effectors in reducing the required dosage of antibiotics against resistant Enterobacteriaceae strains and highlight the need to develop a comprehensive understanding of the underlying mechanisms for the co-action of antimicrobials and QS mediators.

## 1 Introduction

Multidrug-resistant (MDR) bacteria have emerged as a silent pandemic in the world, with the prediction of 10 million deaths annually by 2050 (Tang et al., 2023). MDR Gram-negative bacteria have greatly compromised the clinical efficacy of existing antibiotics. The rise of MDR pathogenic Enterobacteriaceae, particularly *Escherichia coli*, *Salmonella* Typhi, and *Salmonella* Typhimurium, has been fueled by the excessive and irrational use of antibiotics. This has become a pressing public health emergency, particularly in South Asian countries, including Pakistan, which are hot zones for escalating antibiotic resistance (Kang and Song, 2013; Yasmin et al., 2013; Abrar et al., 2018).

Global reports show that extended-spectrum β-lactamase (ESBL)-producing strains of Enterobacteriaceae in clinical isolates exhibit enhanced pathogenicity and environmental fitness, posing a high risk of transmission worldwide due to effective adaptation and better colonization abilities (Park et al., 2012; Ma et al., 2015; Raji et al., 2015; Paitan, 2018). During the last few decades, *E. coli* has emerged as the bacterial pathogen with the highest rate of antibiotic resistance, predominantly, causing treatment failures in urinary tract infections with first-line of antibiotics (Bilal et al., 2021). Gram-negative pathogens are a critical challenge for controlling antimicrobial resistance, costing the United States alone approximately $2.8 billion annually (Shrestha et al., 2018). Numerous epidemiological studies have identified urinary tract infections as the most frequently reported etiology in Pakistan, with diminishing response against last-resort antibiotics due to resistance to third-generation cephalosporins and fluoroquinolones (Bidell et al., 2016).

*Salmonella* Typhi, the causative agent of deadly systemic infection referred to as typhoid fever, has the highest infection rates in Pakistan, as compared to other low- and middle-income countries in South East Asia (Bhan et al., 2005; Karkey et al., 2018; Fatima et al., 2021). Globally, 11.9 to 27.1 million people, including children and the elderly, suffer from typhoid fever each year, with annual mortality rates ranging from 129,000 to 223,000 (Chey et al., 2017). The World Health Organization (WHO) reported extensively drug-resistant *S.* Typhi strains in Pakistan (World Health Organization, 2019), with reduced susceptibility to fluoroquinolone and third-generation cephalosporins, leaving the azithromycin as the drug of choice (Al kraiem et al., 2018). However, the emergence of resistance (>33%) against azithromycin has created a severe therapeutic challenge worldwide (Aziz and Malik, 2018).

*Salmonella* Typhimurium, a major cause of gastroenteritis, is becoming health concern due to its zoonotic nature. Global reports show a multidrug resistance rate of over 90% in this serovar (Qin et al., 2022; Hurtado et al., 2022; Patra et al., 2021). In Pakistan, over 83 million cases of foodborne illnesses are reported annually with 35.2% of multidrug-resistant isolates carrying the *blaTEM-1* gene conferring resistance to beta-lactam antibiotics, including carbapenems and cephalosporins (Abrar et al., 2018b).

Natural products, including those of plant or microbial origin, or their synthetic analogs have been extensively investigated for their potential in combating antimicrobial resistance and constitute a significant proportion of currently used drugs in clinical practice (Newman and Cragg, 2012). Among microbial sources, members of the *Pseudomonas* genus, particularly *P. aeruginosa*, are known for producing bioactive extracellular secondary metabolites that function as signaling molecules to coordinate population behavior in communities, through quorum sensing (QS) (Zhou et al., 2023; Fuqua et al., 2001; Miller and Bassler, 2001; Withers et al., 2001). This intercellular communication system is regulated by autoinducers called N-acyl homoserine lactones (AHLs), mainly used by Gram-negative bacteria; auto-inducing peptides (AIPs), used by Gram-positive bacteria; and AI-2, produced by both (Fuqua et al., 2001; Withers et al., 2001; Mukherjee and Bassler, 2019). Additional signal molecules are *Pseudomonas* quinolone signal (PQS) and its precursor HHQ as well as 4-hydroxy-2-alkyl quinolones (HAQs) (Hays et al., 1945; Wells, 1952), whereas pyocyanin (N-methyl-1-hydroxy phenazine), a terminal signaling molecule, and rhamnolipids serve as virulence factors regulated by QS (Defoirdt, 2018). Pyocyanin and rhamnolipids, have been explored as potent antibacterial compounds (Elshikh et al., 2017; Jameel et al., 2017; Khalid et al., 2022). These novel agents from *P. aeruginosa* have shown *in vitro* activity against a number of the Gram-positive and Gram-negative clinical pathogens (VanDrisse et al., 2021; Defoirdt, 2018).

Despite extensive data on antimicrobial resistance of Enterobacteriaceae strains, much of the research has reported the limited resistance profiles of targeted pathogens, overlooking the rapid acquisition by prevalent strains in clinical settings. There is a critical need for comprehensive studies evaluating the potential of natural or microbial compounds to potentiate the efficacy of conventional antimicrobials, particularly in South Asian regions, where extensive drug resistance of Enterobacteriaceae strains is a significant concern. Addressing this gap requires investigating the non-signaling potency of *P. aeruginosa* QS intermediates, which may contribute to more effective antimicrobial therapies.

Currently, the strategy of combining natural compounds and antimicrobial drugs to enhance or restore the antimicrobial activity spectrum that is no longer effective due to the development of resistance has gained importance as a fast, cost-effective and eco-friendly approach. By using lower but highly effective concentrations of two antimicrobial agents in combination against MDR pathogens, this approach to identifying additive effects has gained importance as a leading pathway to prevent the emergence of antibiotic resistance. Moreover, microbial resistance to these combinations might develop more slowly than when single compounds are involved (Ayaz et al., 2019).

In this study, we investigated the anti-Enterobacteriaceae efficacy of secondary metabolites derived from *P. aeruginosa* strain MC9. The active concentrations of the QS-regulated virulence factors pyocyanin and rhamnolipids as well as the two autoinducers PQS and its precursor HHQ, were determined by MIC assays. Furthermore, we evaluated the synergistic effects of combining these *P. aeruginosa* QS secondary metabolites with conventional antimicrobial drugs.

## 2 Materials and methods

### 2.1 Isolation and identification of *P. aeruginosa*

Twenty-five soil samples were collected from fields at different rice growing sites of NIBGE Faisalabad/Punjab Pakistan. Ten grams of each soil sample were vigorously shaken at 300 rpm with 40 mL of sterile water for 60 min. One mL of each soil suspension was added to 9 mL of Luria Bertani (LB, Oxoid) broth supplemented with 100 μg/L nystatin (Sigma Aldrich) and 100 μg/L cycloheximide (Sigma Aldrich) (Robles-HuÃzar et al., 2017) and incubated at 37 °C for 24 h. A volume of 30 μL from each suspension was spread onto LB agar plates. After overnight incubation at 37 °C, colonies with green pigmentation were selected (Palleroni, 1992). These selected colonies were screened using *Pseudomonas* agar base (Oxoid, PAB, CM0559) supplemented with *Pseudomonas* C-N supplement (SR 102) selective for *P. aeruginosa* and stored in 80% glycerol (Sigma Aldrich) at −20 °C. A vigorous pigment-producing colony was selected and named MC9. Total genomic DNA of this isolate was extracted using the chloroform: isoamyl alcohol method (Thermo Fisher Chemicals) (Sambrook and Russell, 2006). Universal primers PF and PR were used to amplify the 16S rRNA gene (Supplementary Table S1, Serial No. 1) (Awan et al., 2021). The QIA quick Gel Extraction Kit (QIAGEN Sciences, Maryland 20874, USA) was used to clean the amplified PCR product of about 1.5 kb of the 16S rRNA gene. Macrogen, Inc. (Seoul, South Korea) commercially sequenced the cleaned amplified PCR product. Available sequences of bacterial lineages in NCBI were used to align and compare the sequence data using BLAST. The accession number (MK530186) was allocated after submitting the sequences to the NCBI GenBank database.^1^

A phylogenetic tree of isolate MC9 was constructed using Mega 7.0 software based on the TN+G model alignment tool. Sequences were analyzed using the maximum likelihood method (Mirza et al., 2009).

### 2.2 Production of *P. aeruginosa* secondary metabolites

The selected MC9 isolate was inoculated into LB medium (1,000 mL) and incubated with shaking (180 rpm) at 37 °C for 3 days. The bacterial cell pellet was removed by centrifugation of the bacterial culture at 4 °C for 15 min at 10,000 rpm, with a relative centrifugal force (RCF) of 35,000 × *g* at the maximum rotor radius. Liquid chromatography–Mass spectrometry (LCMS) grade chloroform (Thermo Fisher Chemicals) was added to the culture supernatant in a 1:2 ratio and the lower blue organic layer was obtained and evaporated under reduced pressure by rotary evaporation (Heidolph, Schwabach Germany, water bath temperature: 40 °C, rotation speed: 50 rpm, vacuum/pressure 180 mmHg). The resulting residues were dissolved in LCMS grade methanol (Thermo Fisher Chemicals) and stored at −20 °C for further analysis.

### 2.3 Evaluation of the antimicrobial activity of *P. aeruginosa* crude extract and secondary metabolites

#### 2.3.1 Bacterial strains

A total of fourteen clinical isolates (Table 1) were used to screen the antimicrobial activity of the MC9 crude extract. All isolates were morphologically identified by staining characters and reaction in triple sugar iron agar media (Oxoid) slants. For molecular confirmation, the bacterial genomic DNA was extracted using the chloroform-isoamyl alcohol method and genus-specific PCR was performed using previously reported protocols (Saeed et al., 2009). A highly specific *stm* gene fragment was selected for the identification of *S.* Typhimurium (Liora et al., 2013), *fliC* for *S.* Typhi and *uidA* gene for *E. coli.* Final products were confirmed with 1.5% agarose gel electrophoresis (Saeed et al., 2009). The primers used for confirmation of clinical isolates used in the study are given (Serial No. 2–5, Supplementary Table S1).

**Table 1:** Antimicrobial efficacy of *P. aeruginosa* MC9 extract against clinical Enterobacteriaceae strains.

Sr. no.	Bacterial strains	Origin	Antimicrobial resistance profile	*P. aeruginosa* crude methanolic extract concentration per well	Zone of inhibition (mm)
1	*S.* Typhi (18C)	Gastric wound abscess	NA, CIP, PEF, OFX	200 μg/100 μL	17 ± 1
2	*S.* Typhi (22C)	Burn wound	NA, CIP, CAZ, AZM, PEF, OFX	200 μg/100 μL	18 ± 1
3	*S.* Typhi (23C)	Burn wound	NA, CTX, PEF, OFX CFM	200 μg/100 μL	22 ± 1
4	*S.* Typhi (25C)	Burn wound	NA, CIP, AZM, PEF, OFX	200 μg/100 μL	23 ± 1
5	*S.* Typhi (29C)	Diabetic amputation abscess	SXT, NA, ATM, GEN, CRO	200 μg/100 μL	24 ± 1
6	*S.* Typhi (34C)	Diabetic amputation abscess	NA	200 μg/100 μL	18 ± 1
7	*S.* Typhi (39C)	Diabetic amputation abscess	NA, PEF	200 μg/100 μL	16 ± 1
8	*S.* Typhi (AA6)	Diabetic amputation abscess	NA, LEV, CIP, AZM, CTX, PEF, OFX	200 μg/100 μL	13 ± 1
9	*S.* Typhi (AS12)	Diabetic amputation abscess	NA, PEF	200 μg/100 μL	16 ± 1
10	*S.* Typhi (AS26)	Diabetic amutation abscess	NA	200 μg/100 μL	17 ± 1
11	*S.* Typhimurium (W20)	Stool	SXT, NA, ATM, AMC, CRO, CIP. IPM, MEM, DOR, FEP, CAZ, LEV, AK, TOB, TZP, PEF, CTX, CAZ, NOR	200 μg/100 μL	26 ± 1
12	*S.* Typhimurium (W27)	Stool	IPM, CAZ, CIP, LEV, GEN, TIC, AMP, C, PRL, PEF, CTX, CAZ, OFX, NOR	200 μg/100 μL	23 ± 1
13	*E. coli* (SS1)	Abscess	SXT, NA, AMP, C, AZT, AMC, GEN, CRO, CIP	200 μg/100 μL	19 ± 1.5
14	*E. coli* (SS2)	Urine	SXT, NA, AMP	200 μg/100 μL	11 ± 1

SXT, Sulfamethoxazole/trimethoprim; NA, Nalidixic acid; ATM, Aztreonam; AMC, Amoxicillin/clavulanic acid; CRO, Ceftriaxone; CIP, Ciprofloxacin; IPM, Imipenem; MEM, Meropenem; DOR, Doripenem; FEP, Cefepime; LEV, Levofloxacin; AK, Amikacin; GEN, Gentamicin; TOB, Tobramycin; TZP, Tazabactem; PEF, Pefloxacin; CTX, Cefotaxime; CAZ, Ceftazidime; NOR, Norfloxacin; OFX, Ofloxacin; AZM, Azithromycin; AMP, Ampicillin; C, chloramphenicol; PRL, Piperacillin.

#### 2.3.2 Determination of antibiotic resistance profiles of the bacterial isolates

Microbes with non-susceptibility to at least one antimicrobial agent in three or more antimicrobial categories are considered MDR microbes (Rafailidis et al., 2024). Antibacterial susceptibility studies were carried out on the clinical isolates using the Kirby and Bauer disc diffusion technique (CLSI, M100, ED31, 2021) and commercially available antibiotic discs (Oxoid, UK). LB broth containing 0.5 McFarland (MF) turbidity (0.14–0.17 OD_600_) of bacterial culture was spread on Mueller Hinton agar (MHA, Oxoid) plates. Antimicrobial discs were placed on MHA plates about 20 mm apart, inhibition zone diameter was measured after overnight incubation at 37 °C, and results were interpreted following Clinical and Laboratory Standards Institute (CLSI, M100, ED31, 2021) guidelines (Humphries et al., 2021).

#### 2.3.3 Mass spectrometric analysis of bioactive crude extract of *P. aeruginosa* (MC9)

The crude MC9 extract was subjected to electrospray Ionization Mass Spectrometry and tandem mass spectrometry (ESI-MS/MS) analysis using a mass spectrometer (LTQ XL Linear Ion Trap Mass Spectrophotometer, Thermo Scientific, USA), equipped with an ESI source as described previously (Yasmin et al., 2017; Akbar et al., 2019). Briefly, the samples were filter-sterilized and were injected through the direct syringe pump method with a flow rate of 8 μL/min. Samples were scanned at both positive and negative total ion full scan modes (mass scan range *m/z* 50–2,000) with source voltage and capillary voltage of 4.8 kV and 23 V, respectively. Capillary temperature and sheath gas flow (N2) was 350 °C and 30 arbitrary units, in both scan modes. The selected analytes were fragmented in positive and negative ion modes by employing collision-induced dissociation (CID) energy of 35 (percentage of 5 V) or otherwise stated.

#### 2.3.4 Evaluation of antibacterial efficacy of *P. aeruginosa* (MC9) crude extract against the MDR clinical isolates

The MC9 crude extract was evaluated by well diffusion assay for antimicrobial efficacy against selected clinical isolates (Valgas et al., 2007). The inoculum (70 μL) with an optical density of 0.5 MF was uniformly spread on MHA plates and wells were punched in the agar and filled with 100 μL (2,000 μg/mL) test extract. Plates were incubated for 18 h at 37 °C. Methanol (Thermo Fisher Chemicals) was used as a negative control. The zone of inhibition (ZOI) was measured in millimeters (mm) and antibacterial activity was expressed as the average diameter of the ZOI calculated in three independent experimental replicates.

#### 2.3.5 MIC determination of *P. aeruginosa* crude extract and pure compounds

The most prominent compounds selected after MS fingerprinting of crude extract were identified based on standards from Sigma-Aldrich: 5-methyl-1(5H)-phenazinone (210.23, Pyocyanin ≥98% HPLC), 4-hydroxy-2-heptylquinoline (259.34, PQS ≥ 98% HPLC,), 2-heptyl-3-hydroxy-4(1H)-quinolone (243.34, HHQ ≥ 96% HPLC), and rhamnolipids-90%. MICs of MC9 extract and pure compounds were evaluated by “broth microdilution” assay according to the CLSI guidelines, CLSI M100, 31st edition (Humphries et al., 2021) combined with spectrophotometric analysis. Optical density measurements (OD₆₀₀) were recorded using a BioTek Synergy™ H1 microplate reader. MIC values were defined as the lowest concentration of the extract or pure compound that resulted in no measurable/visible increase in optical density compared with the sterile medium control, indicating complete inhibition of visible bacterial growth. Sub-inhibitory concentrations (SICs) were selected as concentrations below the MIC that produced a consistent reduction in optical density relative to the untreated control without complete growth inhibition. OD₆₀₀ was measured after incubation and compared with the untreated growth control. Briefly, stock solutions were serially diluted to make concentrations in the range of 5 mg/mL - 0.01 mg/mL for pyocyanin, PQS, HHQ; 50 mg/mL – 0.3 mg/mL for rhamnolipids and 660 mg/mL – 1 mg/mL for MC9 extract. In the 96-well microtiter plate, one row of 12 wells contained only LB (200 μL/well) as “blank”, whereas another row contained only diluent control (200 μL/well) serially diluted (with LB; v/v) to the concentration corresponding to the respective wells used for MIC determination of tested compounds. The broth culture containing 0.5 MF (1 × 10^8^ CFU/mL) was diluted at a 1:10 ratio to maintain a final inoculum density of 1 × 10^7^ CFU/mL, which was introduced to each of the wells, except the “sterility control wells”. After overnight incubation at 37 °C, the bacterial growth was determined at 600 nm using an ELISA reader (Synergy H1 Biotek microplate reader). The optical density of the “vehicle control” row indicated the maximum concentration of solvent which allow growth of the test bacteria, whereas the “blank” row showing no growth served as a “sterility control” for the procedure and maximum growth is indicated by “growth control lane” containing LB broth and final inoculum. The MIC value is defined as the lowest concentration of the compound that will inhibit the visible growth of a microorganism after overnight incubation (Andrews, 2001).

### 2.4 Synergistic activity of commercial antimicrobials and *P. aeruginosa* secondary metabolites against Enterobacteriaceae isolates

Antimicrobial activity was evaluated using an agar disc diffusion assay with commercially available antibiotic discs (Oxoid) in combination with *P. aeruginosa* secondary metabolites. Details of the antibiotic discs used are presented in Supplementary Tables S4–S7. The 70 μL volume of 0.5 MF bacterial inoculum was evenly spread on MHA plates and left at room temperature for 15 minutes followed by the placement of antimicrobial discs impregnated with tested material. The stock solutions were prepared in 100% methanol with concentrations of 5 mg/mL for pyocyanin, HHQ, PQS, and 50 mg/mL for rhamnolipids. Tested sub-inhibitory concentrations of 10 μg/mL, 50 μg/mL, and 100 μg/mL for pyocyanin, HHQ and PQS, whereas 30 μg/mL, 100 μg/mL, and 500 μg/mL for rhamnolipids were applied on antibiotic discs and placed on the prepared MHA plates. After 18 h incubation at 37 °C ZOI was measured and the percentage (%) increase in ZOI was calculated as (b2-a2)/a2 ×100, where “a” is the ZOI of antibiotic alone and “b” is the ZOI of antibiotic and tested concentration of the pure compound. A combination assay was performed in triplicate and the standard deviation was calculated. All values are expressed as the mean standard error (±) of the mean of triplicate values of the same replicate (Moghaddam et al., 2009).

To confirm the co-activity between *P. aeruginosa* secondary metabolites and antimicrobials, combinations that exhibited strong synergistic effects in the agar disc diffusion assay were selected for further validation. Time-kill assays were subsequently performed using sub-inhibitory concentrations of the antimicrobials and metabolites, and comparative bacterial growth curves were generated to evaluate the interaction effects over time.

### 2.5 Statistics analysis

Statistical comparisons on combination effects by the disc diffusion method were performed using a Student’s *t*-test and Tukey’s *post-hoc* test. A *p*-value of < 0.05 was considered statistically significant.

## 3 Results

### 3.1 Identification and molecular confirmation of *P. aeruginosa*

Among the 25 soil samples, only seven colonies displaying green diffusible pigment production on LB plates were selected. These colonies were subsequently identified using PAB agar, which revealed the characteristic morphology of *P. aeruginosa* with its distinct grape-like odor. Notably, isolate MC9 stood out due to its robust pigmentation. The 16S rRNA gene was amplified and sequenced, with BLAST analysis confirming a 99.85 percent identity with *P. aeruginosa*. A phylogenetic tree based on concatenated 16S rRNA sequences showed that the identified strain clustered with other *P. aeruginosa* isolates (Supplementary Figure S1). The isolate was designated as *P. aeruginosa* MC9 and the 16S rRNA gene sequence was submitted to the GenBank database under accession number MK530186.

### 3.2 Metabolic profiling of crude extract of *P. aeruginosa*

**Figure 1:**
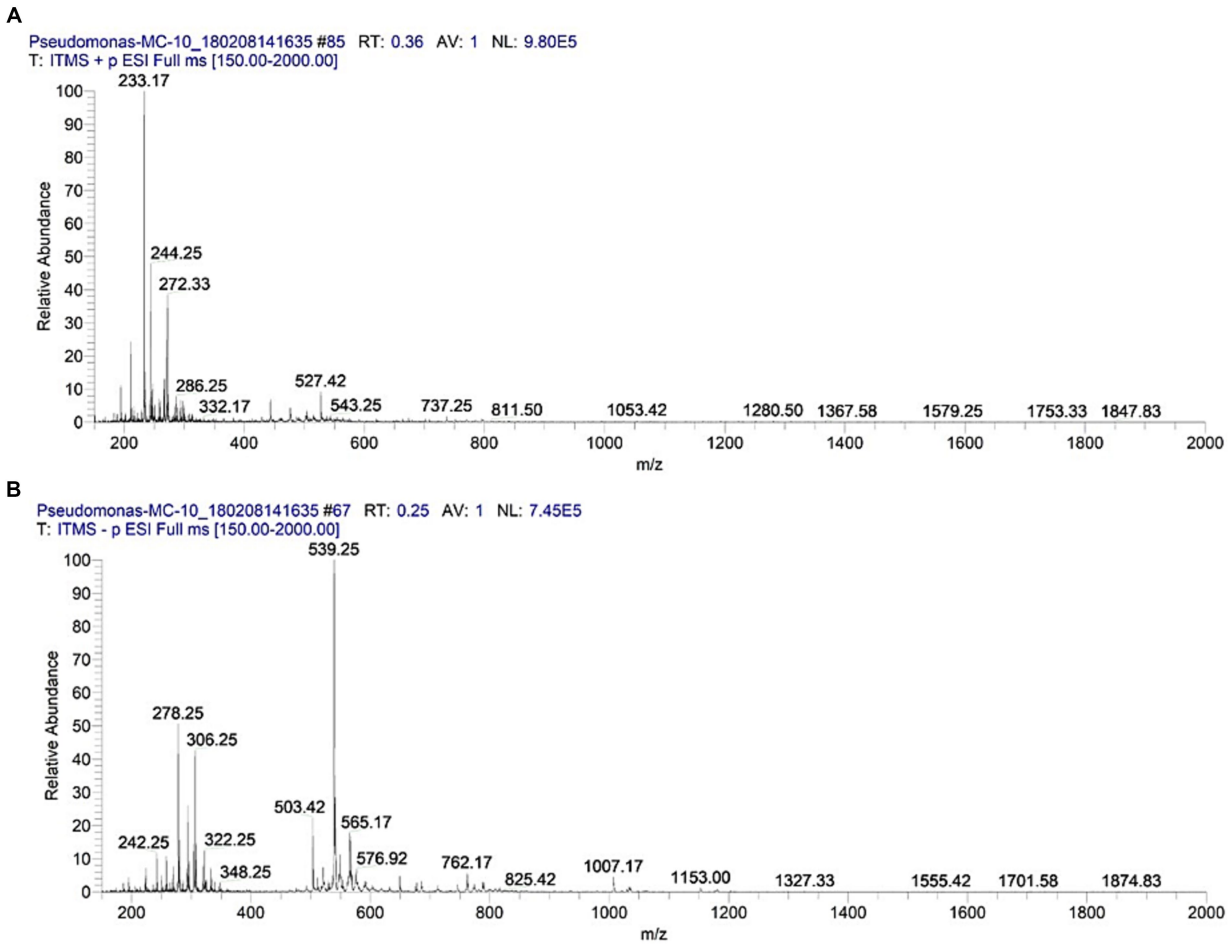
ESI-MS spectra of *P. aeruginosa* MC9 extract. **(A)** ESI-MS spectrum of P. aeruginosa MC9 extract (after 24 h culture) revealed the presence of pyocyanin (5-methyl-1(5H)-phenazinone) at *m/z* 211 and 233 analyzed at positive ion mode. **(B)** ESI-MS spectrum of *P. aeruginosa* MC9 extract (after 72 h culture) revealed the presence of rhamnolipids and their daughter ion species at *m/z* (500-650) analyzed at negative ion mode.

After a 24-h incubation at 37 °C, the crude extract demonstrated the presence of a wide range of metabolites, including phenazines (Figure 1A) along with prominent peaks of HAQs, including QS autoinducers (Figure 1B), whereas after 72 h of incubation, a shift in the metabolic trend occurred, with rhamnolipids becoming the prominent metabolites (Figure 1B). This temporal progression is consistent with the established QS hierarchy in *P. aeruginosa*, in which autoinducers are produced during earlier growth phases and subsequently regulate the production of QS-controlled secondary metabolites. The accuracy of this metabolic profiling was confirmed using MS^n^ and cross-referenced with literature data (Supplementary Table S2).

Pyocyanin (5-methyl-1(5H)-phenazinone) was detected at *m/z* 211.08 [M + H]^+^ and 233.17 [M + Na]^+^. The tandem mass spectrometry of pyocyanin produced 1-hydroxy-phenazine having *m/z* at 196.08 [M + H]^+^ and a related molecule having *m/z* at 183.08 [M + H]^+^ as shown in (Supplementary Figure S2, Supplementary Table S2, Serial No. 1).

ESI-MS/MS investigation of the crude extracts identified a large repertoire of HAQs, with *m/z* spanning from 242 to 298 [M + H]^+^ and from 186 to 332 [M-H]^−^ (Supplementary Table S2, Serial No. 2–6).

All the identified HAQs were classified into distinct sets based on the hydrogen (H), alkyl (R) and hydroxyl (OH) functional groups at two and three positions of the heterocyclic ring as well as the N-oxide group of quinolone nitrogen. Of these series, Series A comprises simpler analogs 4-hydroxy-2-heptylquinoline (HHQs) and 4-hydroxy-2-heptylquinoline N-oxide (HNQs) with hydrogen at 3-position of the heterocyclic ring exhibiting ion peaks at *m/z* at 242.25, 244.25, 256.25, 258.25, 270.25, 272.25, 284.33 and 298.33 [M + H]^+^ and at 186.25, 200.25, 214.25, 240.25, 242.25, 268.25, 270.25, 296.25, 322.25 and 324.25 [M-H]^−^. The members of Series A are differentiated from each other based on the saturation and unsaturation in the R group chain length (Table S2, Serial No 2–14). The dominant analog of Series A was observed at *m/z* 244.25 [M + H]^+^, representing an HHQ. Fragmentation of the parent peak by applying collision-induced dissociation (@CID = 40.0) produced the daughter ion peaks at *m/z* 226.08, 186.08, 172.08, 170.08, 160.08, 159.08 and 146.08 (Supplementary Figure S3, Supplementary Table S2, Serial No. 6). The molecular ion peaks of other analogs of Series A, i.e., at *m/z* 242.17, 256.25, 258.17, 272.25, 284.25 and 298.25 [M + H]^+^ in positive ionization mode and at 186.08, 200.08, 214.17, 240.17, 242.17, 268.25, 270.25, 296.25, 322.25 and 324.25 in negative ionization mode showed a similar pattern of fragmentation upon CID. All of these molecular ion peaks of Series A were extensively analyzed and assigned to the corresponding structures through their detailed tandem mass spectrometry and related literature data (Supplementary Table S2).

Analogous analysis was performed for Series B, C, D, and E, unveiling the structures and fragmentation patterns of various analogs. In Series B, the molecular ion at *m/z* 258.17 [M-H]^−^ representing 3,4-dihydroxy-2-heptylquinoline (HHAQ) exhibited the most prominent peak intensity in negative ion mode. Application of CID led to the gradual loss of CH_2_ groups from the alkyl side chain along with the addition of unsaturation, resulting in the formation of daughter ion peaks at *m/z* at 240.17, 230.25, 214.25, 186.17, 174.08, 173.08, 159.08 and 144.08. These distinct daughter ion peaks confirmed the structure of HHAQ (Supplementary Figure S4, Supplementary Table S2, Serial No. 16). The rest of the molecules from series B were also subjected to MS/MS analysis for structural validation (Supplementary Table S2).

In Series C, the molecular ion peak at *m/z* 286.25 [M-H]^−^, corresponding to 4-hydroxy-3-methyl-2-alkylquinolines N-oxide (HMAQ N-oxide), produced daughter ion peaks with *m/z* at 268.25, 258.25, 242.25, 226.17, 214.17, 200.08, 186.08, 174.08, 173.08, 160.17, 159.08 and 144.08 (Supplementary Figure S5, Supplementary Table S2, Serial No. 17). Two additional analogs of HMAQ N-oxide with octene and octane side chains were also identified through CID, yielding daughter ions at *m/z* 286.25 [M + H]^+^ and *m/z* 288.25 [M + H]^+^, respectively (Supplementary Table S2).

Likewise, in Series D, a polyhydroxy analog having *m/z* at 278 [M-H]^−^ representing 2,3,4-trihydroxy-2-heptylquinoline (HHHAQ) was identified under negative ion mode. Upon applying CID at 35.0, the molecule dissociated into respective daughter ion peaks with *m/z* values at 260.08, 250.06, 242.25, 222.92, 207.08, 194.17, 186.00, 174.08, 158.06, 157.08 and 144.17 [M-H]^−^ (Supplementary Figure S6, Supplementary Table S2, Serial No. 21). Furthermore, the presence of two analogs at *m/z* 236.25 [M-H]^−^ and 332.32 [M-H]^−^ was confirmed through fragmentation into characteristic daughter ions (Supplementary Table S2).

Series E, named as tetrahydroxyl series, exhibited an increased trend. The molecular ion further in was at *m/z* 294.25 [M-H]^−^, with the highest peak intensity and 2-octane chains, corresponded to 2,3,4-trihydroxy-2-heptylquinolines N-oxide (HHHAQ N-oxide). CID (35.0) generated a pattern of daughter ion peaks with *m/z* 258.17, 196.17, 194.08, 184.08, 170.08, 158.06 and 144.08 [M-H]^−^ (Supplementary Figure S7, Supplementary Table S2, Serial No. 23). The remaining analogs of Series E were also systematically analyzed using ESI-MS/MS, which correlated with the expected fragments (Supplementary Table S2).

The results revealed a series of rhamnolipids, including mono-rhamno-di-lipidic and di-rhamno-di-lipidic glycolipid congeners (Supplementary Figure S8, Supplementary Table S2, Serial No. 23). Among these, a mono-rhamnolipid molecule at *m/z* 503.33 was subjected to tandem mass spectrometry resulting in its daughter ion peaks at *m/z* at 339.08, 333.17 and 169.17 [M-H]^−^. Similarly, another analog with *m/z* 677.42 lost one fatty acid chain (C_12_H_23_O_2_^−^ = 198), yielding di-rhamno-mono-lipidic rhamnolipid congener at *m/z* at 479.17. Further fragmentation confirmed ion peaks at *m/z* 333.25 and 169.00 [M-H]^−^, mirroring the fragmentation pattern. Analogous fragmentation patterns were observed for congener ions with *m/z* 649.42 and 762.17 (Supplementary Figure S9, Supplementary Table S2).

### 3.3 Antibacterial efficacy of *P. aeruginosa* (MC9) crude extract against MDR clinical isolates of Enterobacteriaceae

All 14 isolates were identified as Gram-negative bacteria. The distinctive characteristics observed on tryptic soy agar were employed for species identification: *Salmonella* spp. exhibited a yellow slant with a pink butt and a black center accompanied by H_2_S production, whereas *E. coli* showed a yellow slant and yellow butt, along with gas formation, but no H_2_S production. PCR analysis further classified 10 isolates as *S.* Typhi, two as *S.* Typhimurium and the remaining two as *E. coli*. A list of oligonucleotides used for this characterization is given in Supplementary Table S1. In the well diffusion assay, all isolates demonstrated growth inhibition when exposed to MC9 crude extract. The corresponding ZOIs by MC9 crude extract, along with the antimicrobial resistance profiles of each isolates, are given in Table 1. For subsequent investigations comprising MIC determination and synergism assays, three MDR clinical isolates were selected: *S.* Typhimurium-W20, *S.* Typhi-29C and *E. coli*-SS1 with corresponding ZOIs of 26 ± 1, 24 ± 1 and 19 ± 1.5, respectively.

**Figure 2:**
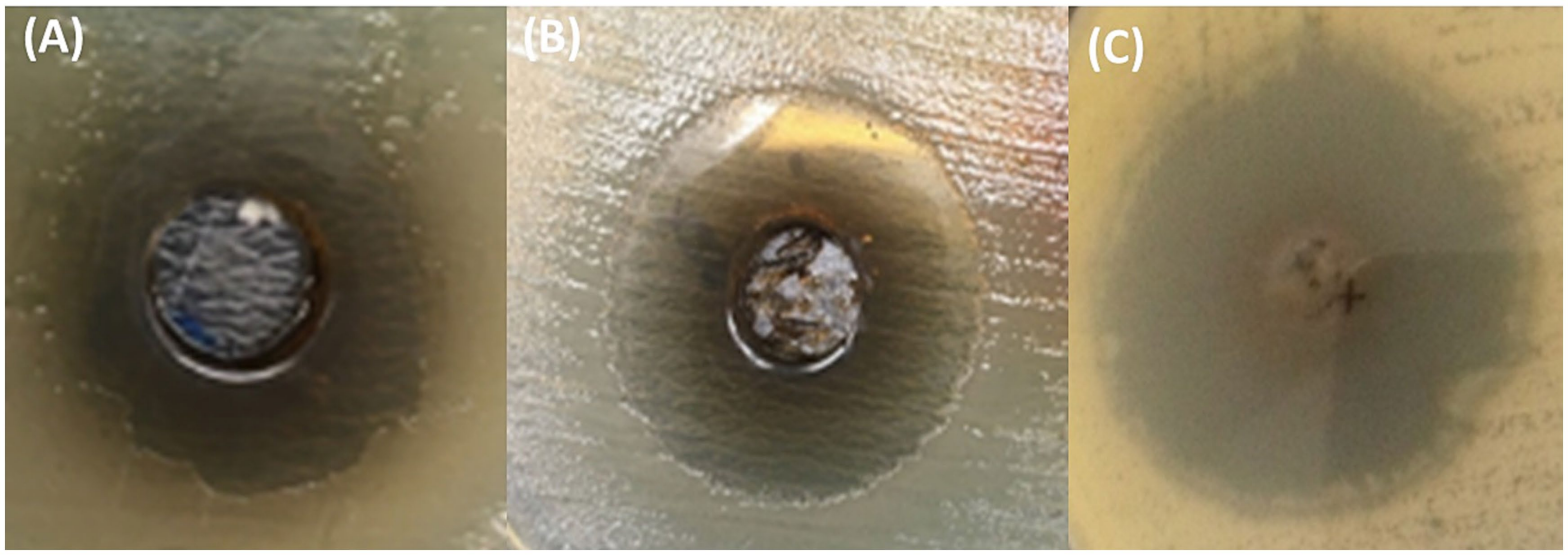
Antimicrobial efficacy of MC9 extract against MDR *Enterobacteriaceae* isolates. **(A)** *E. coli*-SS1, **(B)** *S.* Typhi-29C, and **(C)** *S.* Typhimurium-W20.

The strains were selected based on average ZOI produced by crude MC9 extract against isolates across three independent replicates (Table 1, Figure 2). Based on the results of the agar well diffusion assay, the *E. coli*-SS1 strain exhibited the highest resistance with an inhibition zone of 19 ± 1.5 mm (Figure 2A, Table 1, Serial No. 13) followed by *S.* Typhi-29C with a zone of inhibition of 24 ± 1 mm (Figure 2B, Table 1, Serial No. 5) and *S.* Typhimurium-W20 (Figure 2C, Table 1, Serial No. 11) with an approximately zone of inhibition of 26 ± 1 mm.

A micro broth dilution assay was employed to determine the MIC of the MC9 crude extract, as well as the most dominant secondary metabolites identified in the extract (pyocyanin, rhamnolipids, HHQ, PQS), as confirmed by MS chemical profiling, and the results were interpreted according to CLSI guidelines (Humphries et al., 2021). *S.* Typhi-29C was found to be the most sensitive among the tested clinical isolates, with an MIC of 16 mg/mL. By contrast, *E. coli*-SS1 was found to be the most resistant to MC9 crude extract, with an MIC of 256 mg/mL, while *S.* Typhimurium-W20 showed an MIC of 32 mg/mL (Table 2, Serial No. 1).

**Table 2:** MICs of secondary metabolites of *P. aeruginosa* against MDR Enterobacteriaceae strains.

Sr. no.	*P. aeruginosa* secondary metabolites	Maximum tested concentration (mg/mL)	MDR bacterial strains	MIC (mg/mL)
1	MC9 extract		*S.* Typhi-29C	16
			*S.* Typhimurium*-*W20	32
			*E. coli-*SS1	256
2	Pyocyanin	5	*S.* Typhi-29C	0.1
		5	*S.* Typhimurium*-*W20	0.04
		5	*E. coli-*SS1	0.1
3	Rhamnolipids	50	*S.* Typhi-29C	25
		50	*S.* Typhimurium*-*W20	50
		50	*E. coli-*SS1	50
4	HHQ	5	*S.* Typhi-29C	>5
		5	*S.* Typhimurium*-*W20	>5
		5	*E. coli-*SS1	>5
5	PQS	5	*S.* Typhi-29C	>5
		5	*S.* Typhimurium*-*W20	>5
		5	*E. coli-*SS1	>5

MC9 extract, *P. aeruginosa* crude methanolic extract; HHQ, 2-Heptyl-3-hydroxy-4(1H)-quinolone; PQS, 4-Hydroxy-2-heptylquinoline; MDR, Multidrug resistant.

Among the tested secondary metabolite molecules of *P. aeruginosa* against all MDR Enterobacteriaceae isolates, pyocyanin exhibited the most potent growth inhibitory activity, with MIC values of 100 μg/mL against *S.* Typhi, 40 μg/mL against *S.* Typhimurium and 100 μg/mL against *E. coli* (Table 2, Serial 2). Rhamnolipids showed antimicrobial activity at higher concentrations against all tested isolates. The growth of *S.* Typhi was inhibited at 25 mg/mL, while *S.* Typhimurium and *E. coli* at 50 mg/mL (Table 2, Serial 3). In contrast to pyocyanin and rhamnolipids, the MIC determination of HHQ and PQS showed no antimicrobial efficacy against *S.* Typhi, *S.* Typhimurium, or *E. coli* up to the maximum tested concentration of 5 mg/mL (Table 2, Serial 4, 5).

### 3.4 Evaluation of the synergistic activity of *P. aeruginosa* secondary metabolites with antimicrobials

A disc diffusion assay was used to efficiently determined the synergistic activity of *P. aeruginosa* secondary metabolites in combination with other antimicrobials. (+) indicates synergism, whereas (−) indicates antagonism. Synergism (+) indicates that the combined treatment produced significantly greater growth inhibition than either agent alone. Antagonism (−) denotes reduced growth inhibition /enhanced bacterial growth in the combination compared with individual treatments. Indifferent effects indicate that the combined treatment did not produce a meaningful difference in growth inhibition relative to the single agent.

### 3.5 Pyocyanin combination effects

Sub-inhibitory concentrations of pyocyanin (10 μg/antimicrobial disc, 25 μg/antimicrobial disc and 50 μg/antimicrobial disc), HHQ, PQS (10 μg/antimicrobial disc, 50 μg/antimicrobial disc and 100 μg/antimicrobial disc), and rhamnolipids (30 μg, 100 μg, 500 μg/antimicrobial disc) were used to evaluate the combined effects with nine conventional antimicrobials. The combination combined effects of *P. aeruginosa* QS intermediates with antimicrobials against the tested MDR isolates are presented as mean values of three independent replicates, as depicted in Supplementary Tables S4–S7.

Supplementary Table S4 illustrates the combined effects of pyocyanin with tested antimicrobials against *S.* Typhimurium-W20, *S.* Typhi-29C, and *E. coli*-SS1. Sub-inhibitory concentrations of pyocyanin (10 μg, 25 μg, 50 μg / commercially available antimicrobial disc concentrations) were selected to evaluate the synergistic effects. The growth of *S.* Typhimurium-W20 was unaffected by the combination 10 μg pyocyanin/sulfamethoxazole/trimethoprim 23/1.25 μg, but increasing the pyocyanin dose to 25 μg enhanced the growth inhibiting fold area by 300% with the same antimicrobial. Sulfamethoxazole/trimethoprim 23/1.25 μg did not show a combination effect with any of the three tested concentrations of pyocyanin against *S.* Typhi-29C. However, surprisingly in case of *E. coli*-SS1, this combination strategy exhibited promising results with the combination of 50 μg of pyocyanin and sulfamethoxazole/trimethoprim 23/1.25 μg, increasing the fold area to 116% and thereby restoring the efficacy of the drug as the *E. coli*-SS1 strain was originally resistant to sulfamethoxazole/trimethoprim 23/1.25 μg (Supplementary Table S4, Serial No. 1).

Nalidixic acid (30 μg) demonstrated no antimicrobial augmenting efficacy when combined with 10, 25 and 50 μg pyocyanin against *S.* Typhimurium-W20 and *S.* Typhi-29C. However, a combination of 50 μg pyocyanin increased the inhibition area against *E. coli*-SS1 by 133% (Supplementary Table S4, Serial No. 2).

Combination of ampicillin (10 μg) with 10, 25, or 50 μg of pyocyanin showed an antagonistic effect against *S.* Typhimurium-W20 and *S.* Typhi-29C. By contrast, for *E. coli*-SS1, 10 μg and 25 μg pyocyanin combined with 10 μg ampicillin demonstrated a non-significant effect, whereas further increasing the pyocyanin concentration to 50 μg showed a 125% increase in growth inhibition fold area (Supplementary Table S4, Serial No. 3).

Similar antagonistic trends were observed with chloramphenicol (30 μg) in combination with 10 μg, 25 μg, or 50 μg pyocyanin against *S.* Typhimurium-W20 and *S.* Typhi-29C. However, for *E. coli*-SS1, 10 μg and 25 μg pyocyanin combined with chloramphenicol 30 μg demonstrated no significant effect, whereas increasing the pyocyanin concentration to 50 μg resulted in a 225% increase in growth inhibition fold area (Supplementary Table S4, Serial No. 3).

Combining pyocyanin at sub-inhibitory concentration of 10 μg with aztreonam (10 μg) enhanced the antibacterial efficacy against *S.* Typhimurium-W20 by 16%. However, increasing pyocyanin to 25 μg did not amplify this effect. Intriguingly, the combination 50 μg pyocyanin with10 μg aztreonam showed an indifferent effect, counteracting the augmenting efficacy of lower pyocyanin doses. For *S.* Typhi-W20, the trend was totally different, as 10 μg and 50 μg pyocyanin/10 μg aztreonam both demonstrated antimicrobial potentiating efficacy of 11% and 23%, respectively, whereas 25 μg of pyocyanin showed indifference. In case of *E. coli*-SS1, all three tested sub-inhibitory concentrations of pyocyanin with 30 μg aztreonam showed antagonistic effect (Supplementary Table S4, Serial No. 5).

The combination of 30 μg amoxicillin clavulanic acid with 10 μg, 25 μg, or 50 μg of pyocyanin showed antagonistic effects against *S.* Typhimurium-W20, but for *S.* Typhi-29C and *E. coli*-SS1, this combination was totally indifferent with no antimicrobial augmenting potential (Supplementary Table S4, Serial No. 6).

Combining sub-inhibitory concentrations of 10 μg and 25 μg pyocyanin with gentamicin (30 μg) showed favorable results for *S.* Typhimurium-W20, increasing the growth inhibition area by 21% and 25%, respectively. However, increasing the pyocyanin concentration to 50 μg showed only a 9.7% increment. For *S.* Typhi-29C, both 10 μg and 25 μg pyocyanin with gentamicin (30 μg) augmented growth inhibition by 199 and 201%, respectively, whereas 50 μg pyocyanin exhibited only 7% drug augmenting efficacy. The results were very different for *E. coli*-SS1, as all tested combinations demonstrated antagonistic effects (Supplementary Table S4, Serial No. 7).

The combination of 30 μg ceftriaxone with 10 μg pyocyanin showed a synergistic effect, increasing the growth inhibiting area by 32%. Increasing pyocyanin dose to 25 μg or 50 μg showed indifferent combination effects. In the same way, ceftriaxone (30 μg) with pyocyanin at 10 μg, 25 μg or 50 μg was ineffective for *S.* Typhi-29C and *E. coli*-SS1 (Supplementary Table S4, Serial No. 8).

The combination of ciprofloxacin (5 μg) with pyocyanin was studied for *S.* Typhimurium. The most effective synergy was observed at a concentration of 10 μg pyocyanin, resulting in a 40% increase in the growth inhibiting fold area. However, combinations with sub-inhibitory concentrations of 25 μg and 50 μg of pyocyanin exhibited ineffective outcomes (Supplementary Table S4, Serial No. 8).

**Figure 3:**
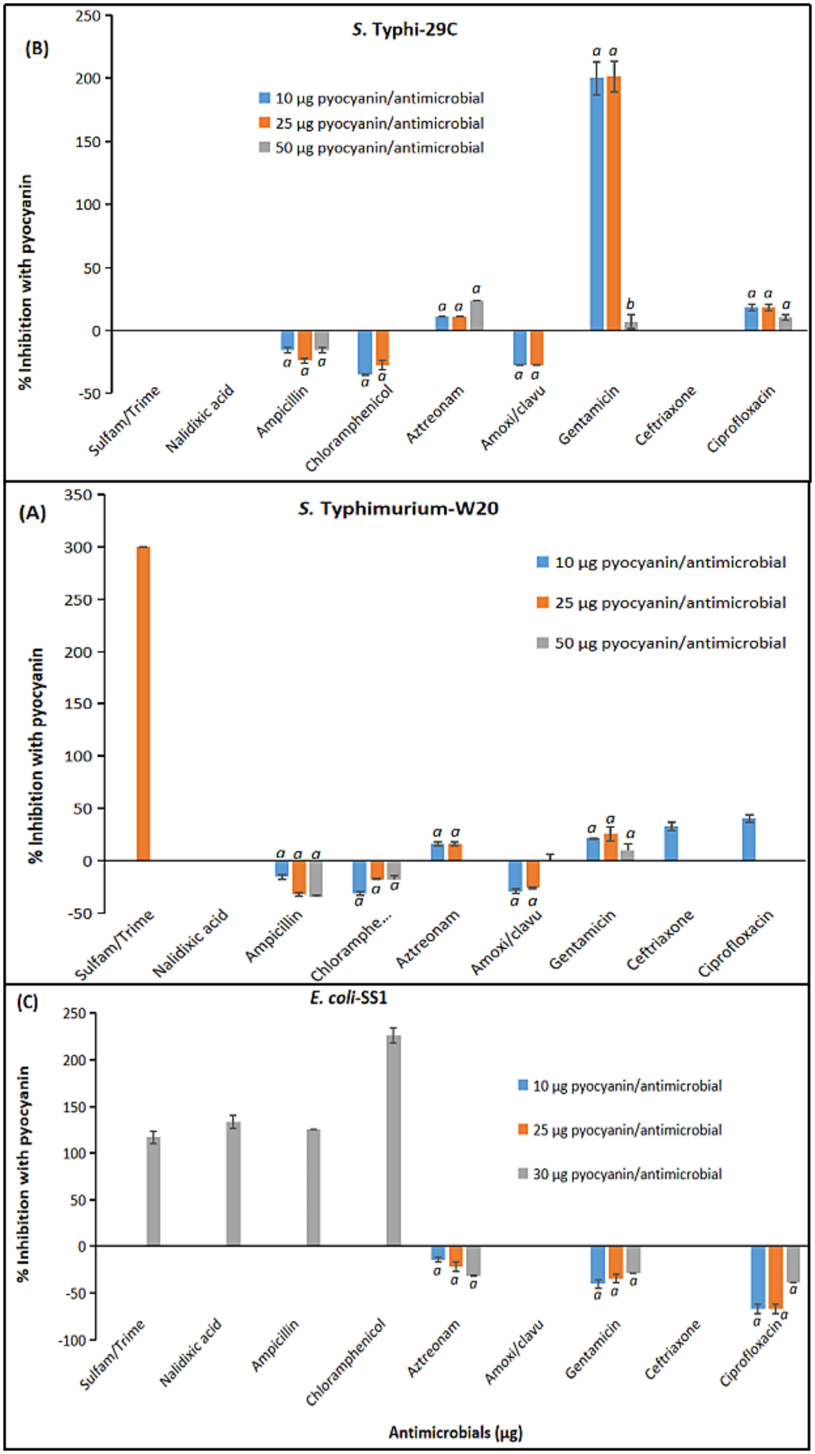
Pyocyanin combination effects with antimicrobials against MDR *Enterobacteriaceae* isolates. **(A)** *S.* Typhimurium-W20. **(B)** *S.* Typhi-29C. **(C)** *E. coli*-SS1. Statistical comparison of experimental groups was performed using one-way ANOVA followed by Tukey’s *post hoc* test. Groups not sharing the same letter **(a, b, c)** differ significantly (*p* < 0.05). Error bars indicate mean +/− standard error (*n* = 3). Only data with mean values >2% are presented.

A comprehensive comparison of the combination effects of antimicrobials with three sub-inhibitory concentrations of pyocyanin is presented in Figure 3 (a, b, c), providing a visual representation of the varying combination outcomes across different antimicrobial agents and concentrations.

### 3.6 Rhamnolipids combination effects

Supplementary Table S5 shows the combined effects of rhamnolipids with tested antimicrobials against *S.* Typhimurium-W20, *S.* Typhi-29C, *E. coli*-SS1. Three sub-inhibitory concentrations of rhamnolipids, 30 μg, 100 μg, and 500 μg per disc, were chosen to evaluate the combination effects.

Sulfamethoxazole/trimethoprim (23/1.25 μg) showed no synergistic effect with any of the tested sub-inhibitory concentrations of rhamnolipids against the clinical isolates of Enterobacteriaceae (Supplementary Table S5, Serial No. 1).

Nalidixic acid (30 μg) demonstrated no antimicrobial augmenting efficacy with 30 μg, 100 μg, and 500 μg rhamnolipids against *S.* Typhimurium-W20 and *E. coli*-SS1. However, for *S.* Typhi, the combination of 500 μg rhamnolipids with nalidixic acid increased the inhibition area by 36% (Supplementary Table S5, Serial No. 2).

The combination of ampicillin (10 μg) with rhamnolipids (30 μg) showed an antagonistic antimicrobial effect against *S.* Typhimurium-W20, whereas increasing the concentration of rhamnolipids to 100 μg increased the growth inhibiting area by 11%. However, the combination with 500 μg rhamnolipids was ineffective for enhancing the antimicrobial efficacy. The clinical isolate *S.* Typhi-29C was not affected by the combination of ampicillin (10 μg) with 30 μg rhamnolipids, whereas other sub-inhibitory concentrations of 100 μg and 500 μg showed synergistic antimicrobial effects of 6% and 4%. Conversely, in the case of *E. coli*-SS1, only the combination of ampicillin (10 μg) and 500 μg rhamnolipids exhibited a substantial enhancement of antimicrobial efficacy to 36%, whereas the other two sub-inhibitory concentrations of rhamnolipids (30 μg and 100 μg) showed indifferent combination results (Supplementary Table S5, Serial No. 3).

All tested combinations of chloramphenicol (30 μg) and rhamnolipids (30 μg, 100 μg, 500 μg) showed antagonistic effects against *S.* Typhimurium-W20. Conversely, these combinations yielded non-significant effects for *S.* Typhi-29C. For *E. coli*-SS1, only the combination of chloramphenicol (30 μg) and 500 μg rhamnolipids showed a substantial 77% increase in inhibition area. The other two sub-inhibitory concentrations of rhamnolipids (30 μg and 100 μg) with 30 μg of chloramphenicol proved ineffective in enhancing the growth inhibiting efficacy (Supplementary Table S5, Serial No. 4).

In case of *S.* Typhimurium-W20, combining rhamnolipids (30 μg) with aztreonam (10 μg) resulted in decreased antibacterial efficacy, indicating an antagonistic antimicrobial combination. However, for *S.* Typhi the combination of 30 μg rhamnolipids and 10 μg aztreonam enhanced the growth inhibiting fold area to 13%. Further increase in the rhamnolipids concentration showed an indifferent effect, effectively reversing the augmenting efficacy of 30 μg rhamnolipids (Supplementary Table S5, Serial No. 5).

The combination of amoxicillin clavulanic acid (30 μg) with sub-inhibitory concentrations of rhamnolipids (30 μg, 100 μg, 500 μg) demonstrated indifferent antimicrobial effects against *S.* Typhimurium-W20 and *S.* Typhi-29C. However, for *E. coli*-SS1, the combination of amoxicillin clavulanic acid (30 μg) and rhamnolipids (30 μg) exhibited a substantial 205% synergistic antimicrobial efficacy. The other two sub-inhibitory concentrations of rhamnolipids combined with amoxicillin clavulanic acid showed no antimicrobial augmenting potential (Supplementary Table S5, Serial No. 6).

The combination of gentamicin (30 μg) with sub-inhibitory concentrations of rhamnolipids (30 μg, 100 μg and 500 μg) showed antagonistic antimicrobial effects against *S.* Typhimurium-W20, whereas these combinations yielded indifferent results against *S.* Typhi-29C and *E. coli*-SS1 (Supplementary Table S5, Serial No. 7).

The combinations of ceftriaxone (30 μg) and 30 μg rhamnolipids for *S.* Typhimurium-W20 was synergistic, with the increase in growth inhibiting area by 34%. Further increasing the rhamnolipid concentration to 100 μg and 500 μg resulted in indifferent combination effects. Conversely, all these combinations showed indifferent antimicrobial effects against *S.* Typhi-29C.

Surprisingly, all three combinations of ceftriaxone (30 μg) and rhamnolipids exhibited approximately the same degree of enhancement of 106% in the antimicrobial fold area against *E. coli*-SS1 (Supplementary Table S5, Serial No. 9).

**Figure 4:**
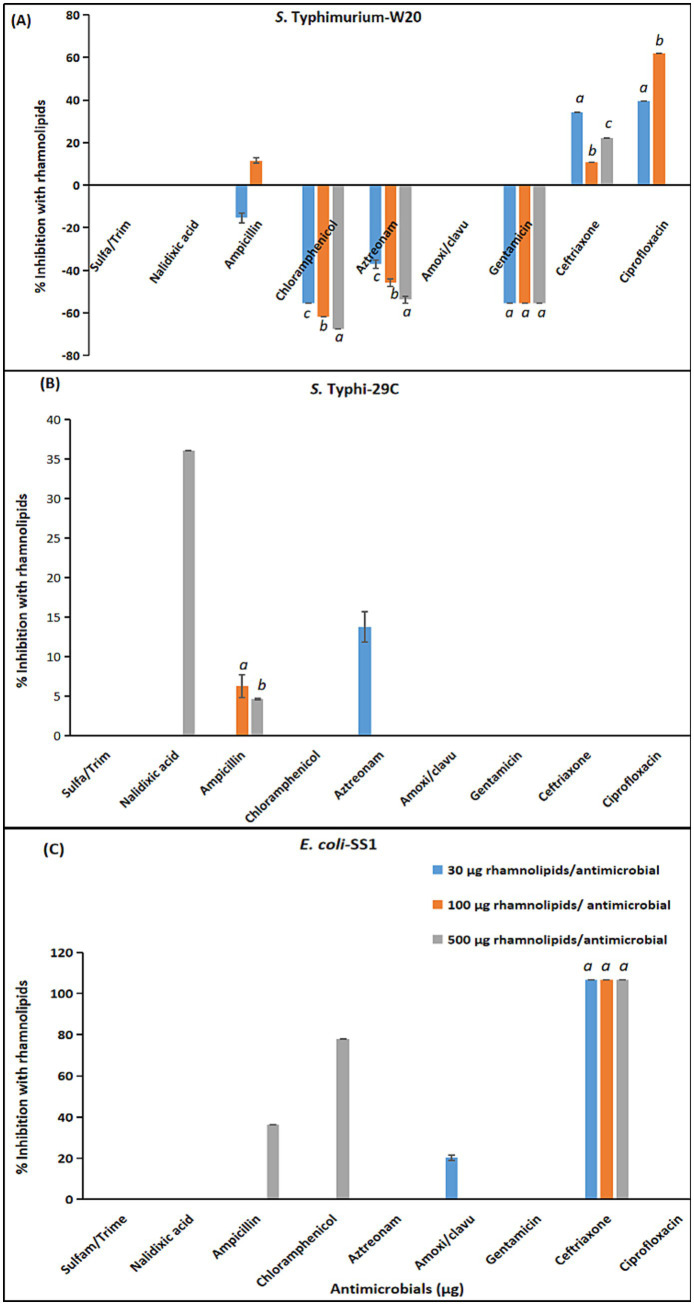
Rhamnolipids combination effects with antimicrobials against MDR *Enterobacteriaceae* isolates. **(A)** *S.* Typhimurium-W20. **(B)** *S.* Typhi-29C. **(C)** *E. coli*-SS1. Statistical comparison of experimental groups was performed using one-way ANOVA followed by Tukey’s post hoc test. Groups not sharing the same letter (a, b, c) differ significantly (*p* < 0.05). Error bars indicate mean +/− standard error (*n =* 3). Only data with mean values >2% are presented.

A visual representation of the comparative combination effects of antimicrobials with three sub-inhibitory concentrations of rhamnolipids is given in Figure 4 (a, b, c).

### 3.7 HHQ combination effects

Supplementary Table S6 demonstrates the combination effects of HHQ with tested antimicrobials against the selected isolates. HHQ enhanced the antibacterial efficacy of antibiotics against *S.* Typhimurium-W20, with augmentation percentages ranging from 10% to a substantial 257%.

Sulfamethoxazole/Trimethoprim (23/1.25 μg) failed to show combination effect with 10 μg, 50 μg and 100 μg sub-inhibitory concentrations of HHQ against the tested clinical Enterobacteriaceae isolates (Supplementary Table S6, Serial No. 1).

Nalidixic acid (30 μg) followed the same trend as observed with sulfamethoxazole/trimethoprim, showing no antimicrobial augmenting efficacy with 10 μg, 50 μg and 100 μg HHQ against *S.* Typhimurium-W20, *S.* Typhi-29C and *E. coli*-SS1 (Supplementary Table S6, Serial No. 2).

The combination of ampicillin (10 μg) and HHQ (10 μg, 50 μg, 100 μg) demonstrated non-significant antimicrobial effects for both *S.* Typhimurium-W20 and *S.* Typhi-29C. However, a remarkable result was noted for the combination of ampicillin (10 μg) and 100 μg HHQ, which significantly enhanced the antimicrobial efficacy against the clinical isolate of *E. coli* to 257% (Supplementary Table S5, Serial No. 2), although the combination was ineffective at 10 μg or 50 μg of HHQ concentrations.

For all tested combinations of chloramphenicol (30 μg) and HHQ (10, 50, 100 μg), antagonistic effects were observed against *S.* Typhimurium-W20. Interestingly, for *S.* Typhi-29C, the combination of chloramphenicol (30 μg) with HHQ (10 μg and 50 μg) augmented the drug efficacy to 36 and 56%, respectively, whereas enhancing the HHQ dose to 100 μg decreased the inhibition fold area by 26% as compared to 50 μg of HHQ. For *E. coli*-SS1, growth remained largely unaffected by the combinations, except for the synergistic combination of chloramphenicol (30 μg) and 100 μg HHQ, which substantially enhanced the antimicrobial efficacy by 77% (Supplementary Table S6, Serial No. 4).

In case of *S.* Typhimurium, combining HHQ at a concentration of 10 μg with aztreonam (30 μg), the antibacterial efficacy was potentiated by 11%, whereas increasing the HHQ concentration to 50 μg did not further support this synergistic effect. Surprisingly, 100 μg of HHQ with 30 μg of aztreonam diminished the synergistic effect for *S.* Typhimurium-W20. For *S.* Typhi, the combinations of aztreonam (30 μg) with 10 μg, 50 μg, and 100 μg HHQ demonstrated approximately equal synergistic effects of up to 11%. The trend remained similar for *E. coli*-SS1, with approximately the same degree of antimicrobial augmenting efficacy observed across all three tested combinations (Supplementary Table S6, Serial No. 5).

The combination of 30 μg amoxicillin clavulanic acid with 10 μg and 500 μg of HHQ demonstrated a synergistic antimicrobial effect for *S.* Typhimurium-W20 with an inhibition percentage of 14%, whereas the growth inhibition was indifferent to the combination with 100 μg HHQ. The synergistic combination with 10 μg and 50 μg of HHQ showed 44% increase in fold area inhibition for *S.* Typhi-29C. Increasing the HHQ dose to 500 μg only slightly enhanced the growth inhibiting area by 10%. *E. coli*-SS1 was strongly inhibited by the combinations of amoxicillin clavulanic acid (30 μg) and HHQ. The combinations with 10 μg, 50 μg, 100 μg HHQ increased the% inhibition area by 141, 136 and 141%, respectively (Supplementary Table S6, Serial No. 6).

The combination of gentamicin (30 μg) with selected concentrations of HHQ (10 μg, 50 μg and 100 μg) showed ineffective antimicrobial effects against *S.* Typhimurium-W20 and *S.* Typhi-29C. However, in case of *E. coli*- SS1, only the synergistic combination of gentamicin (30 μg) and 100 μg HHQ exhibited a 30% enhancement in the growth inhibition fold area (Supplementary Table S6, Serial No. 7).

Combining 30 μg ceftriaxone with 10 μg and 50 HHQ was synergistic for *S.* Typhimurium-W20, with the increase in growth inhibiting area by 28%. Elevating the HHQ concentration to 100 μg did not further support to increase the growth inhibiting area. Moreover, the combination 30 μg ceftriaxone/500 μg HHQ showed antimicrobial synergism up to 10%. For *S.* Typhi-29C, the combinations of 30 μg ceftriaxone with 10 μg and 50 μg HHQ demonstrated synergistic activity of approximately 23%, and with 500 μg a good synergistic combination increasing the growth inhibiting fold area by 49%. However, the combination with 10 μg HHQ was ineffective for *E. coli*-SS1. Intriguingly, the combination of ceftriaxone (30 μg) with 50 μg HHQ exhibited an unexpectedly high level of inhibition of approximately 89%. Increasing the HHQ concentration to 100 μg only increased the fold area to 11% (Supplementary Table S6, Serial No. 8).

The synergistic combination of ciprofloxacin (5 μg) and HHQ (10 μg) enhanced the fold area by 89% for *S.* Typhimurium-W20, whereas a further increase in the concentration to 50 μg or 100 μg just increased the growth inhibiting area to 39% in both cases. However, the combinations of ciprofloxacin (5 μg) with 10 μg, 50 μg, and 100 μg HHQ were ineffective for *S.* Typhi-29C and *E. coli*-SS1 (Supplementary Table S6, Serial No. 8).

**Figure 5:**
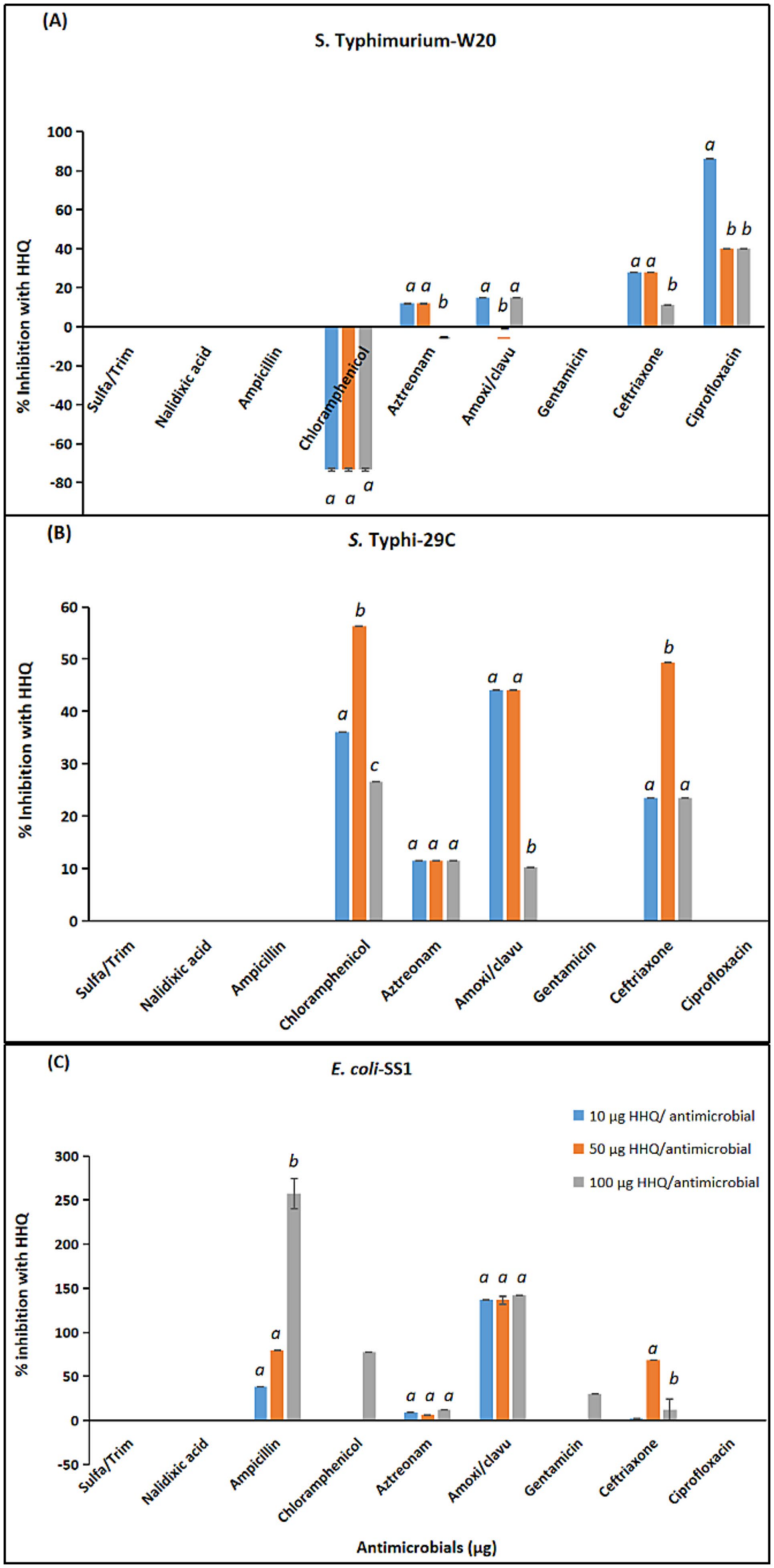
2-Heptyl-3-hydroxy-4(1H)-quinolone (HHQ) combination effects with antimicrobials against MDR *Enterobacteriaceae* isolates: **(A)** *S.* Typhimurium-W20S; **(B)** *S.* Typhi-29C; **(C)** *E. coli*-SS1. Statistical comparison of experimental groups was performed using one-way ANOVA followed by Tukey’s post hoc test. Groups not sharing the same letter (a, b, c) differ significantly (*p* < 0.05). Error bars indicate mean +/− standard error (*n =* 3). Only data with mean values >2% are presented.

Comparative combination effects of antimicrobials with three sub-inhibitory concentrations of HHQ against MDR clinical isolates of *S.* Typhimurium-W20, *S.* Typhi-29C and *E. coli*-SS1 are presented in Figure 5 (a, b, c).

### 3.8 PQS combination effects

Supplementary Table S6 provides a comprehensive comparison of the combination effects of tested antimicrobials with three randomly chosen concentrations (10, 50, 100 μg/disc) of PQS against MDR clinical isolates. PQS exhibited the potential to enhance the antibacterial efficacy of antimicrobials against the tested isolates, ranging from 8% to 108%.

Sulfamethoxazole/trimethoprim (23/1.25) μg as well as nalidixic acid (30 μg) showed no significant combination effect with 10 μg, 50 μg or 100 μg sub-inhibitory concentrations of PQS against the tested clinical isolates of Enterobacteriaceae (Supplementary Table S6, Serial No. 1 and Serial No. 2).

For the combination of ampicillin (10 μg) and PQS (10 μg), a 31% increase in inhibition area was observed for *S.* Typhimurium-W20, but the combination of ampicillin (10 μg) with 50 μg and 100 μg PQS only moderately enhanced the fold area to 14%. For *S.* Typhi, all these combinations were antagonistic. Against the clinical isolate *E. coli*-SS1, all three combinations resulted in indifferent outcomes (Supplementary Table S6, Serial No. 3).

All tested combinations of chloramphenicol (30 μg) with PQS (10 μg, 50 μg, 100 μg) showed indifferent effects against the *S.* Typhimurium isolate. The scenario shifted for *S.* Typhi-29C, where the combination with 10 μg PQS augmented the drug efficacy to 26%. However, increasing the PQS dose to 50 μg and 100 μg only increased the inhibition fold area to 8%. *E. coli*- SS1 growth remained unaffected by all these combinations (Supplementary Table S6, Serial No. 4).

Combining PQS (10 μg) with aztreonam (30 μg) against *S.* Typhimurium-W20 potentiated the antibacterial efficacy by 23%, whereas increasing the PQS concentration to 50 μg and 100 μg did not support further enhancement beyond 23%. In case of the *S.* Typhi strain, the combination of 30 μg of aztreonam with 10 μg and 50 μg PQS demonstrated approximately equal synergistic effects, both around 35%. Increasing the PQS concentration to 100 μg only slightly enhanced the inhibition fold area to 11%. Moreover, *E. coli*-SS1 demonstrated indifferent results against all tested combinations (Supplementary Table S6, Serial No. 5).

The combination amoxicillin clavulanic acid (30 μg) with10 μg of PQS demonstrated a synergistic antimicrobial effect with 30% synergistic inhibition and increased dose of PQS to 50 μg increased the fold area by 47%. However, the synergistic combination with 100 μg of PQS only increased the inhibition fold area by 14% (Supplementary Table S6, Serial No. 6).

Combining gentamicin (30 μg) with sub-inhibitory concentrations of PQS (10 μg, 50 μg and 100 μg) exhibited antagonistic antimicrobial effects against *S.* Typhimurium and *S.* Typhi, whereas in case of *E. coli*, all combinations of gentamicin (30 μg) with PQS were ineffective for increasing the growth inhibition area (Supplementary Table S6, Serial No. 7).

The combination of ceftriaxone (30 μg) with PQS (10 μg, 50 μg, 100 μg) for *S.* Typhimurium was synergistic with the increase in growth inhibiting area by 22%. However, all the tested combinations were antagonistic for *S.* Typhi, and indifferent results were observed against *E. coli* (Supplementary Table S6, Serial No. 8).

The synergistic combinations of ciprofloxacin (5 μg) with PQS (10 μg and 100 μg) for *S.* Typhimurium enhanced the inhibition fold area to 39%, but the combination with 50 μg PQS surprisingly enhanced the growth inhibition to 85%. A similar trend was observed for *S.* Typhi, as the combination of ciprofloxacin (5 μg) and PQS (50 μg) provided the highest synergistic combination, increasing the growth inhibition to 93%, whereas the other two combinations with 10 μg and 100 μg PQS increased the inhibitory effect by 45 and 73%, respectively. However, the combinations of ciprofloxacin (5 μg) with PQS were ineffective for *E. coli* (Supplementary Table S6, Serial No. 9).

**Figure 6:**
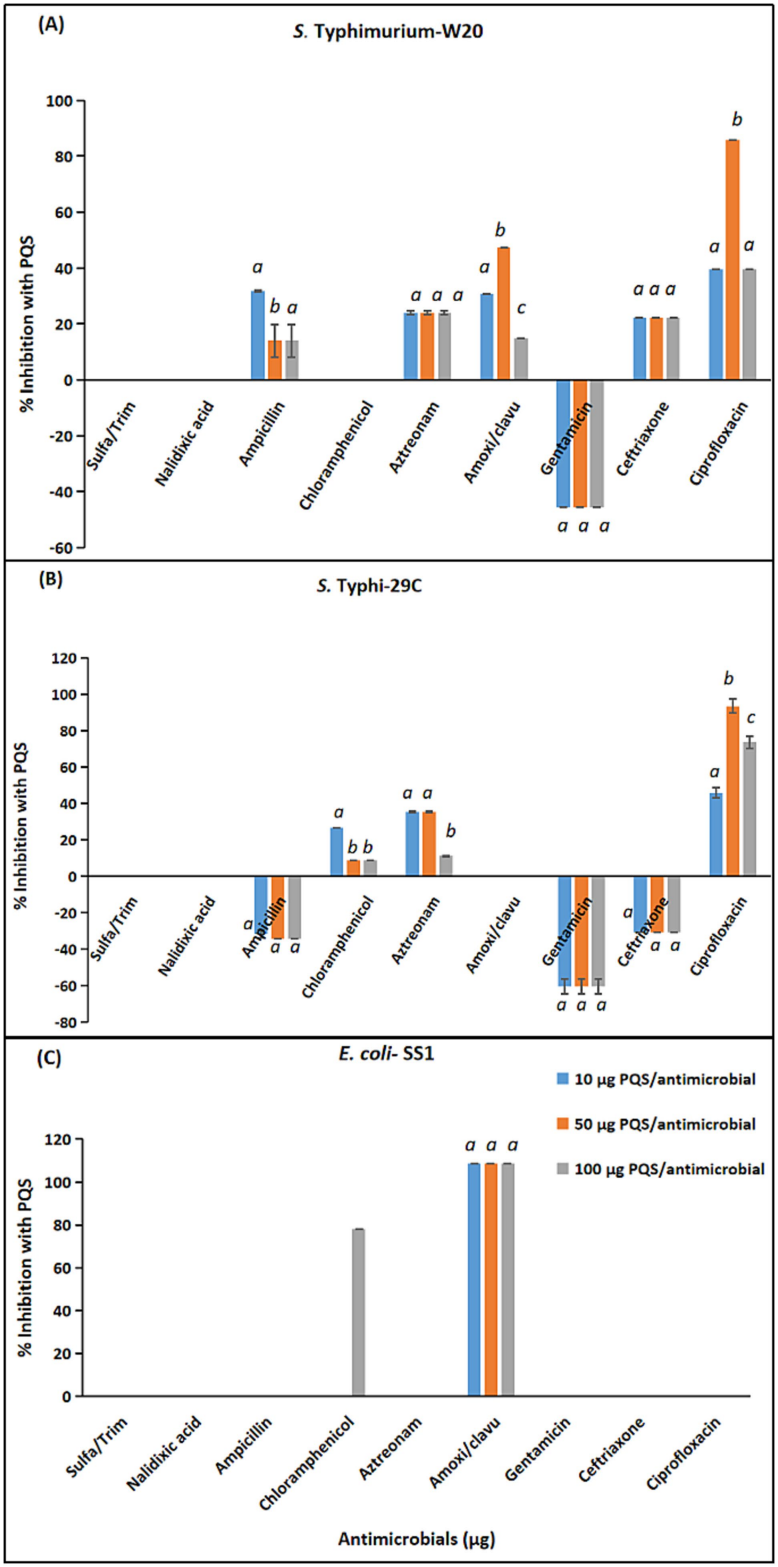
4-Hydroxy-2heptylquinoline (PQS) combination effects with antimicrobials against MDR *Enterobacteriaceae* isolates. **(A)** *S.* Typhimurium-W20. **(B)** *S.* Typhi-29C. **(C)** *E. coli*-SS1. Statistical comparison of experimental groups was performed using one-way ANOVA followed by Tukey’s post hoc test. Groups not sharing the same letter (a, b, c) differ significantly (*p* < 0.05). Error bars indicate mean +/− standard error (*n =* 3). Only data with mean values >2% are presented.

Comparative combination effects of antimicrobials with three sub-inhibitory concentrations of PQS against *S.* Typhimurium, *S.* Typhi and *E. coli* are given in Figure 6 (a, b, c).

### 3.9 Growth curve assay

To substantiate the observed synergistic efficacy of sub-inhibitory concentrations of antimicrobials, as previously observed by agar disc diffusion assay, a comprehensive time-dependent growth curve assay was performed. This assay provided an in-depth evaluation of the impact of selected concentrations of pyocyanin, rhamnolipids, HHQ, and PQS, both alone and in combination with sub-inhibitory concentrations of antimicrobials, on the growth of *S.* Typhi-29C, *S.* Typhimurium-W20, and *E. coli*-SS1.

**Figure 7:**
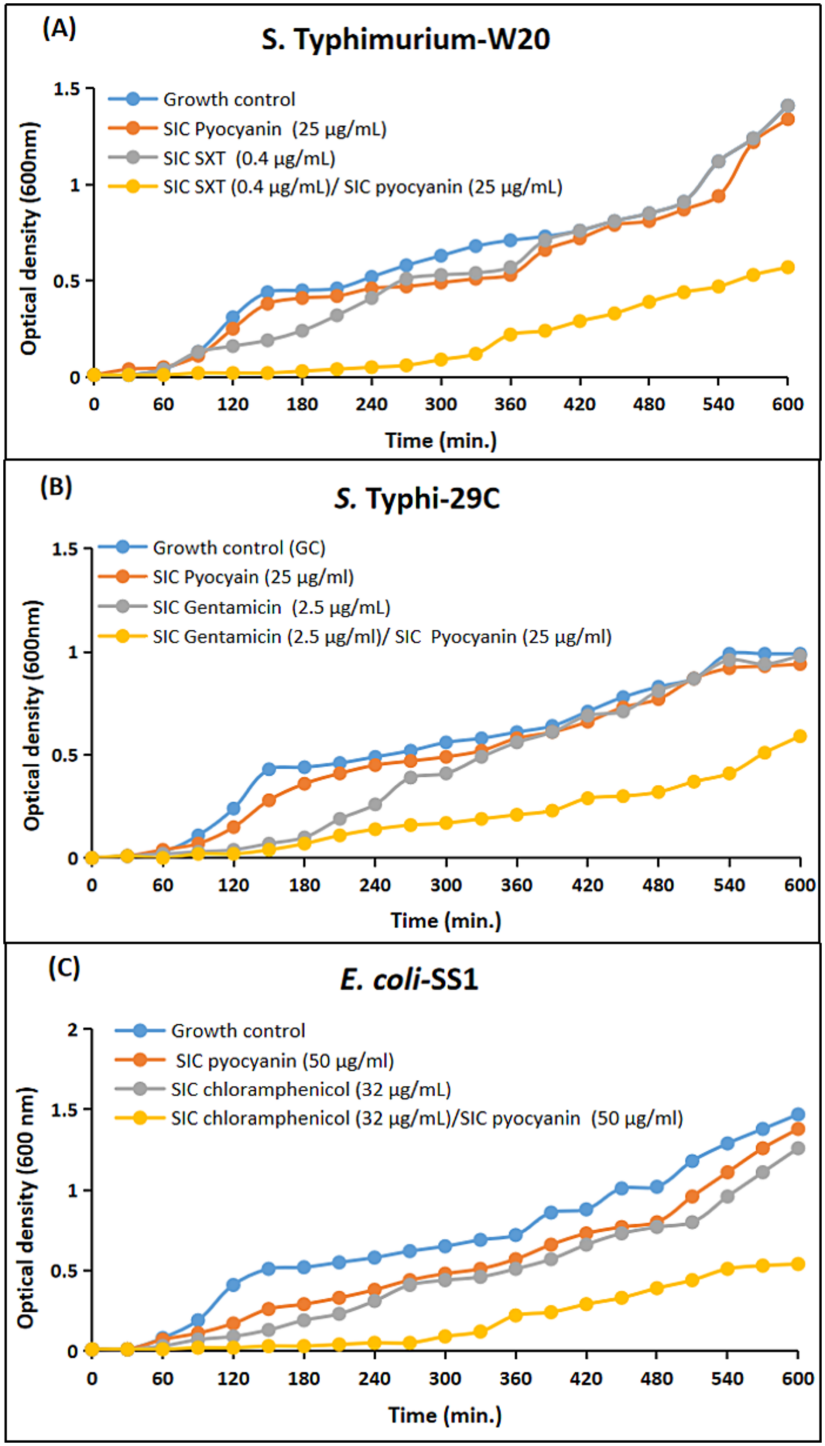
Comparative growth curves representing the drug augmenting efficacy of pyocyanin with sub-inhibitory concentration (SIC) of antimicrobials against Enterobacteriaceae clinical isolates. **(A)** *S.* Typhimurium-W20; blue: Growth control; red: Microbial growth with pyocyanin (25 μg/mL); grey: Microbial growth with SIC of sulfamethoxazole/trimethoprim (SXT, 0.4 μg/mL); yellow: Microbial growth with both SIC of SXT (0.4 μg/mL) and SIC of pyocyanin (25 μg/mL). **(B)** *S.* Typhi-29C; blue: Growth control; red: Microbial growth with pyocyanin (25 μg/mL); grey: Microbial growth with SIC of gentamicin (2.5 μg/mL); yellow: Microbial growth with both SIC of gentamicin (2.5 μg/mL) and SIC of pyocyanin (25 μg/mL). **(C)** Growth curves representing the drug potentiating efficacy of pyocyanin against *E. coli*-SS1; blue: Growth control; red: Microbial growth with pyocyanin (50 μg/mL); grey: Microbial growth with SIC of chloramphenicol (32 μg/mL); yellow: Microbial growth with SIC of chloramphenicol (32 μg/mL) and SIC of pyocyanin (50 μg/mL).

**Figure 8:**
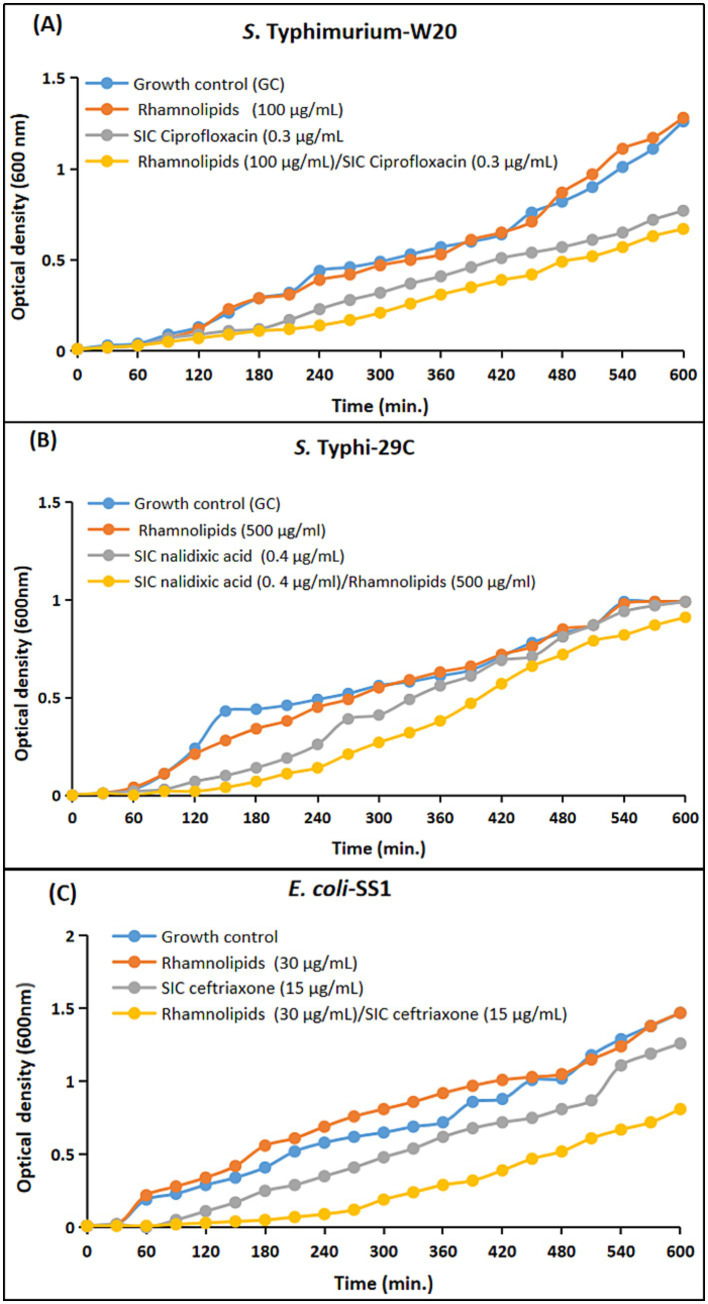
Comparative growth curves representing the drug augmenting efficacy of rhamnolipids with sub-inhibitory concentration (SIC) of antimicrobials against Enterobacteriaceae clinical isolates. **(A)** *S.* Typhimurium-W20: Blue: Growth control; Red: microbial growth with rhamnolipids (100 μg); Grey: microbial growth with SIC of ciprofloxacin (0.3 μg); Yellow: with both SIC of ciprofloxacin (0.3 μg) and rhamnolipids (100 μg/mL). **(B)** *S.* Typhi-29C: Blue: Growth control; Red: microbial growth with rhamnolipids (500 μg); Grey: microbial growth with SIC of nalidixic acid (0.4 μg); Yellow: with both SIC of nalidixic acid (0.4 μg) and rhamnolipids (500 μg/mL). **(C)** *E. coli*-SS1: Blue: Growth control; Red: microbial growth with rhamnolipids (30 μg); Grey: microbial growth with SIC of ceftriaxone (15 μg); Yellow: microbial with both SIC of ceftriaxone (15 μg) and rhamnolipids (30 μg/mL).

**Figure 9:**
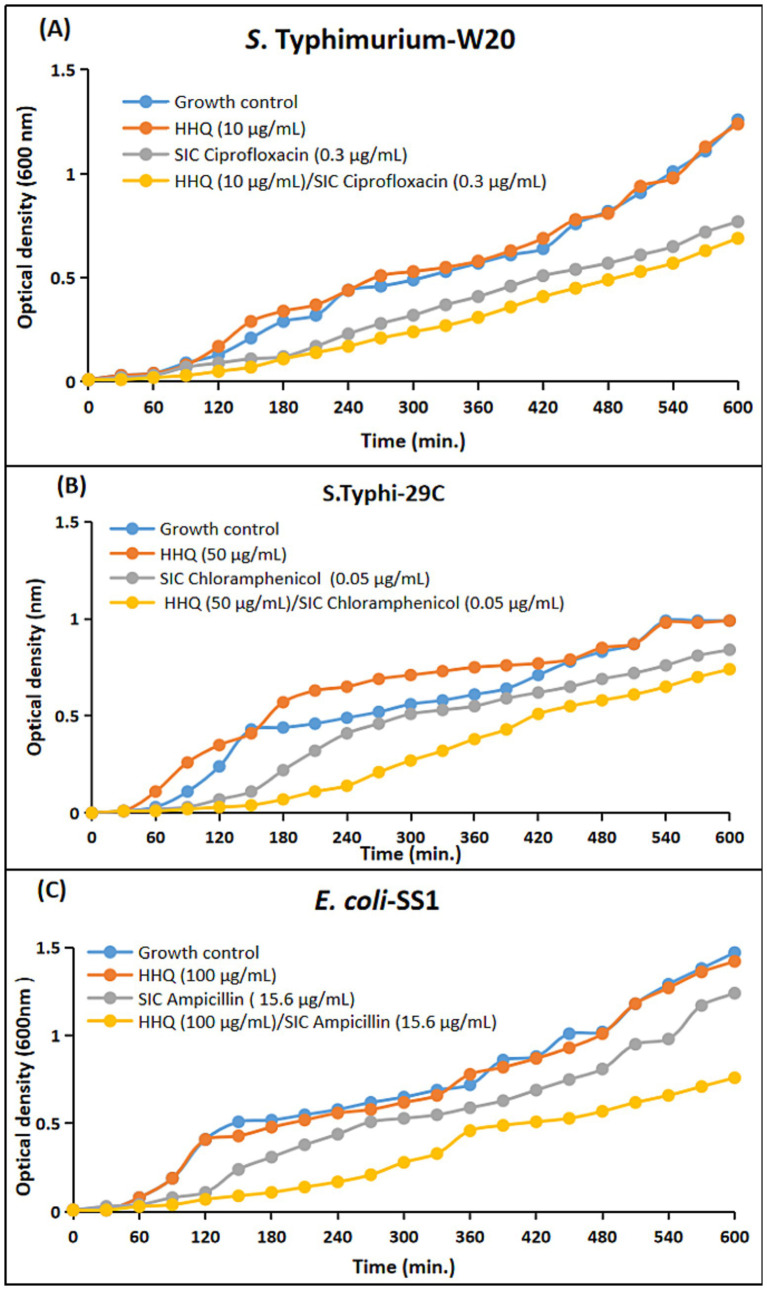
Comparative growth curves representing the drug augmenting efficacy of 2-heptyl-3-hydroxy-4(1*H*)-quinolone (HHQ) with sub-inhibitory concentration (SIC) of antimicrobials against *Enterobacteriaceae* clinical isolates. **(A)** *S.* Typhimurium-W20: Blue: Growth control; Red: Microbial growth with HHQ (10 μg/mL); Grey: Microbial growth with SIC of ciprofloxacin (0.3 μg); Yellow: Microbial growth with both SIC of ciprofloxacin (0.3 μg) and HHQ (10 μg/mL). **(B)** *S.* Typhi-29C: Blue: Growth control; Red: Microbial growth with HHQ (50 μg/mL); Grey: Microbial growth with SIC of chloramphenicol (0.05 μg); Yellow: Microbial growth with both SIC of ciprofloxacin (0.3 μg) and HHQ (50 μg/mL). **(C)** *E. coli*-SS1: Blue: Growth control, no ampicillin (15.6 μg)/HHQ (100 μg/mL); Red: Microbial growth with HHQ (100 μg/mL); Grey: Microbial growth with SIC of ampicillin (15.6 μg); Yellow: Microbial growth with both SIC of ampicillin (15.6 μg) and HHQ (100 μg/mL).

**Figure 10:**
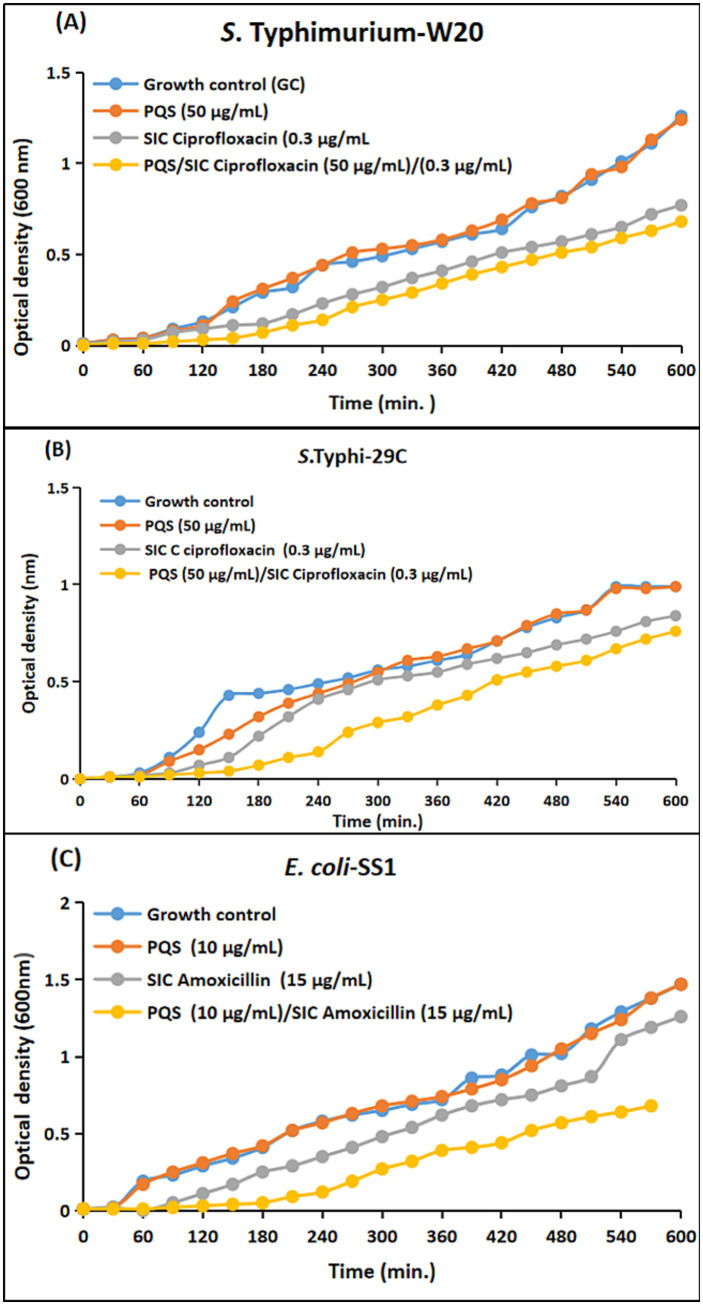
Comparative growth curves representing the drug augmenting efficacy of 4-hydroxy-2heptylquinoline (PQS) with sub-inhibitory concentration (SIC) of antimicrobials against Enterobacteriaceae clinical isolates. **(A)** *S.* Typhimurium-W20; Blue: Growth control; Red: microbial growth with PQS (50 μg/mL); Grey: microbial growth with both SIC of ciprofloxacin (0.3 μg); Yellow: microbial growth with SIC of ciprofloxacin (0.3 μg) and PQS (50 μg/mL). **(B)** *S.* Typhi-29C; Blue: Growth control; Red: microbial growth with PQS (50 μg/mL); Grey: microbial growth with SIC of ciprofloxacin (0.3 μg); Yellow: microbial growth with both SIC of ciprofloxacin (0.3 μg) and PQS (50 μg/mL). **(C)** *E. coli*-SS1; Blue: Growth control; Red: microbial growth with PQS (10 μg/mL); Grey: microbial growth with SIC of amoxicillin (15 μg); Yellow: microbial growth with SIC of ciprofloxacin (15 μg) and PQS (10 μg/mL).

For the generation of time-response curves, the growth dynamics of the clinical strains were monitored at 37 °C, with OD_600_ measurements recorded at hourly intervals spanning up to 10 h. The analysis of the curves showed that all the clinical strains in the panel exhibited reduced growth patterns upon the simultaneous administration of the selected combinations as compared to the individual antibacterial efficacies. In case of pyocyanin, the growth of the clinical strains was notably suppressed when subjected to the combination regimen. This suppression was in stark contrast to the growth patterns observed when pyocyanin was administered alone, where comparatively less inhibition was observed. However, the scenario changed when considering rhamnolipids, HHQ, and PQS. These compounds, when employed in isolation, displayed minimal antibacterial efficacy. This phenomenon was visually portrayed in Figures 7–10, wherein the growth curves in response to various treatments were plotted. The curves clearly illustrated the growth-inhibiting effects of the selected combinations, further validating the potential synergistic interactions observed through earlier assays.

## 4 Discussion

The *Pseudomonas* genus is best known for the production of potent bioactive QS modulators, which facilitate the behavioral synchronization of bacterial cell populations (Wratten et al., 1977; Lazdunski et al., 2004; Supong et al., 2016; Saalim et al., 2020). These QS mediators are consequential for a microbial signaling cascade (Sifri, 2008; Whiteley et al., 2017; Barriuso et al., 2018), displaying antibiotic and anti-biofilm activities across a wide spectrum of bacterial species. This provides significant benefits to the bacteria in host colonization, the formation of biofilms, defense against competitors, and adaptation to environmental changes (Tan et al., 2020). The QS autoinducers, including PQS and its precursor HHQ, mediate bacterial communication and regulate the biofilm formation. Although some QS-regulated metabolites may exhibit antibiofilm activity against other microorganisms, within the microbial community, they generally promote biofilm development (Lee and Zhang, 2015). Over the past two decades, QS-based intervention has gained importance as a first-line therapeutic approach against microbial infections (Hassan and Fridovich, 1980; Brehm-Stecher and Johnson, 2003; Inoue et al., 2004; Kaufmann et al., 2005; Jabra-Rizk et al., 2006; Qazi et al., 2006; Biswas et al., 2009; Cerca et al., 2012; John et al., 2016; Singh et al., 2016; Noto et al., 2017). In this study, the significant bioactive potential of selected QS effectors from the crude extract of *P. aeruginosa* isolate MC9 was explored against MDR *Salmonella* and *E. coli* isolates.

During the initial 24-h growth period of *P. aeruginosa* strain MC9, MS spectra of crude extracts showed the predominant pyocyanin peaks, whereas in the next 24 h, dominant production of HAQs and rhamnolipids was observed in *P. aeruginosa* strain MC9. This closely mirrors previously described QS-controlled metabolic patterns (El-Sheshtawy and Doheim, 2014; Yasmin et al., 2017). Notably, the MC9 crude extract was active against all tested clinical isolates of the three tested species, though this effect was species-specific and correlated with the antimicrobial resistance profile of each strain. Pyocyanin demonstrated the strongest antimicrobial activity. Furthermore, the clinical isolates showed enhanced sensitivity to antimicrobials when combined with pyocyanin, aligned with previous reports (Ghazi and Kahya, 2021; Dhairh and Al-Azawi, 2022). The bioactivity of pyocyanin can be attributed to its formation of reactive oxygen species (−O_2_ and H_2_O_2_) within the target cells, causing disruptions in the electron transport chain (Sudhakar et al., 2015) and inhibiting NADPH pathways, thereby inducing oxidative stress in bacterial cells (Hall et al., 2016). This oxidative stress compromises cell function and renders bacterial cells more susceptible to antimicrobials, thereby contributing to synergism or enhanced antibiotic activity. Moreover, free radicals impact gene expression, potentially supporting antibiotic efficiency (Abdul-Lateef et al., 2017). On the other hand, the thin peptidoglycan layer in the cell wall of Gram-negative bacteria (El-Shouny et al., 2011; Paltansing, 2015; Dhairh and Al-Azawi, 2022) facilitates easy penetration of pyocyanin, potentially boosting the anti-Gram-negative activity of antibiotics (Willey et al., 2009; Das et al., 2014; Zaman et al., 2017). Notably, the efficiency of pyocyanin as a virulence factor of *P. aeruginosa* has increased over time to combat the competition in the increasingly resistive environment (Stover et al., 2000). The bioactivity of pyocyanin is exquisitely sensitive to tested concentrations, as observed in the current study and similar results that were reported previously (Ghazi and Kahya, 2021; Dhairh and Al-Azawi, 2022). Pyocyanin compromises the electron transport chain function, rendering it effective against both Gram-positive and Gram-negative bacteria, with differences in MICs attributable only to the antibiotic susceptibility of the target bacterium (Hassan and Fridovich, 1980; Kaufmann et al., 2005; Biswas et al., 2009; Noto et al., 2017).

By contrast, rhamnolipids displayed activity against all tested bacteria, albeit with a comparatively higher MIC, up to 50 mg/mL, as compared to pyocyanin. The exciting fact was that they have the ability to boost the activity of tested antimicrobials at concentrations significantly lower than the globally accepted MICs (Rivardo et al., 2011; do Valle Gomes and Nitschke, 2012; Sotirova et al., 2012; Hobley et al., 2015). While rhamnolipids are appreciable natural biosurfactants (Bordoloi and Konwar, 2008; Pornsunthorntawee et al., 2008; El-Sheshtawy and Doheim, 2014), their antimicrobial efficacy, particularly against Gram-negative bacteria due to the presence of an outer lipopolysaccharide barrier, has been limited (Bharali and Konwar, 2011). However, they have exhibited efficacy against Gram-positive bacteria and fungi (El-Shouny et al., 2011; Das et al., 2014). This antimicrobial characteristic can be attributed to the insertion of rhamnolipid acyl tails into the cell membrane, leading to the disruption of cytoskeletal components and the imbalance of cytoplasmic elements through leakage. Additionally, their hydrophobic and hydrophilic ends insert into the cell membrane, creating disruptive holes and altering the ultrastructure of the cell (Pearson et al., 1997; Bharali et al., 2013). Furthermore, rhamnolipids increase cell permeability to antibiotics, serving as the fundamental reason behind their synergistic behavior with tested antibiotics (Shusterman et al., 2021). Based on our results, *E. coli* was found to be the most responsive bacterium among the tested isolates to combination therapy with rhamnolipids (Krause et al., 2016). Being the most resistant strain, the enhancement of cell membrane permeability for efficient antibiotic uptake emerges as a promising strategy (Wood et al., 2018; Shusterman et al., 2021). Interestingly, rhamnolipids reduced cell adhesion within biofilms of *S. enteritidis* and *E. coli*, thus weakening the protective barrier and paving the way for potentiated antibiotic activity (Rivardo et al., 2011; do Valle Gomes and Nitschke, 2012; Hobley et al., 2015). On a separate note, it was observed that rhamnolipids were not as effective as pyocyanin in combination results because of the differential mechanisms and machinery of the bacteria targeted by various classes of antimicrobials (Ng and Li, 2017). The efficient entry of antimicrobials induced by rhamnolipids does not guarantee enhanced antibiotic action in target cells, as the combination results are potentially influenced by differences in methodology, bacterial strains, resistance profiles, and rhamnolipids concentrations (Shusterman et al., 2021).

In concordance with prior studies, our data show that HHQ and PQS did not show any growth inhibitory activity against Enterobacteriaceae isolates under the tested conditions. This suggests that these QS effectors lack antagonistic action and are unlikely to serve as antimicrobials against Gram-negative bacteria (Tashiro et al., 2010; Szamosvari et al., 2019; Klaus et al., 2020). By contrast, HHQ was a potent antimicrobial and antibiofilm agent against many Gram-positive bacteria (Wratten et al., 1977; Machan et al., 1992; Lacey et al., 1995; Debitus et al., 1998; Hoffman et al., 2006; Rattanachuay et al., 2011; Nguyen et al., 2016). The bacteriostatic activity of HHQ was observed to be species-specific but also sensitive to structural modifications (Szamosvari et al., 2019; Ramos et al., 2020). Moreover, the N-oxides of HAQs showed a broader spectrum of antimicrobial activity as compared to their reduced counterparts. The bioactivity of HAQs hinges on factors such as degree of the unsaturation, alkyl chain length and branching, with HAQs exhibiting high activity against Gram-positive bacteria and relatively weaker antimicrobial potential against Gram-negative bacteria, supporting the results of the ongoing study (Szamosvari and Bottcher, 2017; Piochon et al., 2020; Saalim et al., 2020). To the best of our knowledge, the present study represents the first report exploiting the antimicrobial activity of QS terminal effector PQS and its precursor HHQ against *S.* Typhi.

Our results demonstrated synergism between PQS, HHQ, and many of the tested antimicrobials. Neither of these QS signals showed any inhibitory activity against the tested Enterobacteriaceae strains up to concentrations of 5 mg/mL. Nevertheless, the exceedingly low concentrations at which synergism was observed when combined with antibiotic MICs is noteworthy. PQS was previously found to directly bind to hundreds of proteins within cells leading to altered transcriptional profiles of related genes (Hodgkinson et al., 2016; Baker et al., 2017; Dandela et al., 2018). These changes might be beneficial for the efficiency of antimicrobials offering a plausible reason for the synergistic activity of PQS combined with antibiotics. Interestingly, the Gram-negative external membrane barrier possesses no resistance to PQS as it mediates the formation of outer membrane vesicles observed in *E. coli* (Tashiro et al., 2010). HHQ, on the other hand, repressed the motility and biofilm formation of broad range of pathogens including Gram-negative bacteria. Moreover, both Gram-positive and Gram-negative pathogens changed their surface-associated phenotypes upon exposure to PQS and its precursor HHQ (Mooij et al., 2011). These findings suggest that QS intermediate, including PQS/HHQ, have significant roles extending beyond inter-cell signaling and can be analyzed via scaffold engineering leading to highly effective combination therapies with antibiotics, even at very low inhibitory concentrations. In addition, there is a need to investigate the non-signaling and signaling roles of HHQ and PQS, offering promising avenues for combating the emerging resistant Gram-negative pathogens.

The results of the current study demonstrated the potential of using *P. aeruginosa* QS intermediates including pyocyanin, HAQs, and rhamnolipids as sensitivity boosters in antimicrobial therapies, especially against MDR strains of *Salmonella* and *E. coli.* However, the species-specific antimicrobial potential and concentration-dependent synergistic potential observed in this study suggests that further experiments are required to evaluate detailed drug-potentiating mechanisms to optimize therapeutic strategies.

Despite the observed synergistic effects, the safety of *P. aeruginosa* derived metabolites requires careful consideration, as this species is highly pathogenic and many of its QS-regulated metabolites are linked to virulence (Lee and Zhang, 2015). In this study, these compounds were evaluated *in vitro* at defined concentrations as isolated molecules rather than as products of live bacteria. The results indicate their potential use as antibiotic adjuvants (Chung et al., 2023) highlighting the need for further studies on toxicity, host–pathogen interactions, and safe molecular optimization prior to any clinical application.

## 5 Conclusion

The study highlights the potential secondary metabolites derived from *P. aeruginosa* MC9 effective enhancers of antimicrobial activity against MDR *Salmonella* and *E. coli* strains. The MC9 crude extract, particularly its QS effectors, exhibited significant antimicrobial activity and enhanced the efficacy of conventional antimicrobials, with pyocyanin demonstrating the most pronounced synergistic potential. This research demonstrates both the antimicrobial effects and the synergistic potential of these QS molecules against MDR Enterobacteriaceae isolates. Notably, these findings are based on *in vitro* evaluations at defined concentrations, and further quantitative synergy and safety assessments will be essential to establish therapeutic feasibility. Nevertheless, these findings offer promising insights for future drug development strategies, particularly in the context of enhancing the efficacy of existing antibiotics against resistant bacterial strains.

## Data Availability

The original contributions presented in the study are included in the article/Supplementary material, further inquiries can be directed to the corresponding authors.

## Footnotes

1
https://www.ncbi.nlm.nih.gov/genbank/

## Glossary

NIBGE-C: National Institute for Biotechnology and Genetic Engineering College
PIEAS: Pakistan Institute of Engineering and Applied Sciences
MDR: Multidrug resistance
ESBLs: Extended-spectrum ß-lactamases
QS: Quorum sensing
AHLs: N-acyl-L-homoserine lactones
HAQs: 4-hydroxy-2-alky quinolones
HQNO: N-oxide derivatives
PQS: *Pseudomonas* quinolone signal
LC–MS: Liquid chromatography - mass spectrometry
MIC: Minimum inhibitory concentration
LB: Luria Bertani
PAB: *Pseudomonas* agar base
UV: Ultraviolet
DNA: Deoxyribonucleic acid
RNA: Ribonucleic acid
NCBI: National Center for Biotechnology Information
BLAST: Basic Local Alignment Search Tool
CID: collision-induced dissociation
TSI: Triple Sugar Iron
MF: McFarland standard
MHA: Mueller-Hinton agar
CLSI: Clinical & Laboratory Standards Institute
ZOI: Zone of inhibition
mm: millimeter
HPLC: High-performance liquid chromatography
OD: Optical density
ESI-MS/MS: Electrospray Ionization Mass Spectrometry and tandem mass spectrometry
MW: Molecular weight
HHAQ: 3,4-dihydroxy-2-heptylquinoline
HHHAQ: 2,3,4-trihydroxy-2-heptylquinoline
SXT: Sulfamethoxazole/ Trimethoprim
NADPH: Nicotinamide adenine dinucleotide phosphate reduced form
