# Supplementary material for: Unlocking the potential of Pseudomonas aeruginosa QS intermediates as antimicrobial synergists against three multidrug-resistant enteric bacteria
Source: Front Microbiol. 2026 Mar 5;17:1726554. doi: 10.3389/fmicb.2026.1726554 (PMC12999905; doi:10.3389/fmicb.2026.1726554)
Supplement: Supplementary file 1 [file Data_Sheet_1.pdf]

Supplementary Material

Unlocking the Potential of *Pseudomonas aeruginosa* QS intermediates as Antimicrobial Synergists Against Three Multidrug-Resistant Enteric Bacteria

Saba Kiran[1,3], Alaa S. Alhegaili[2], Noura Al-Dayan[2], Zubera Naseem[1], Waqar Siddique[1], Itra Ayoub[1], Shoaib Iqbal[1], Sobia Jabeen[1], Fazl-e-Habib[1], Saman Taj[1], Ashfaq Hussain[1], Rizwan Bashir[1], Yasra Sarwar[1], Aamir Ali[1], Waqar Rauf[1*], Georg Jander[3], Mazhar Iqbal[1*].

[1]Health Biotechnology Division, National Institute for Biotechnology and Genetic Engineering College, Pakistan Institute of Engineering and Applied Sciences (NIBGE-C, PIEAS), Faisalabad-38000, Punjab, Pakistan.

[2]Department of Medical Laboratory, College of Applied Medical Sciences, Prince Sattam bin Abdulaziz University, P.O Box 422, Alkharj 11942, Kingdom of Saudi Arabia

[3]Boyce Thompson Institute, Ithaca, New York 14853, USA

*** Correspondence:**
1- Mazhar Iqbal
hamzamgondal@gmail.com

2- Waqar Rauf
wadhamite@gmail.com

Table S1. Oligonucleotides used in this study for confirmation of clinical isolates

Sr No.	Bacteria	Genes		Primer sequences (5′-3′)	Amplicon size (bp)	References
1	*P. aeruginosa*	*PaSS*	F<br>R	GGGGGATCTTCGGACCTCA<br>TCCTTAGAG TGCCCACCG	956	[1]
2	*E. coli*	*uidA*	F<br>R	ATCACCGTGGTGACGCATGTCGC<br>CACCACGATGCCATGTTCATCTGC	486	[2]
3	*S.* Typhi	*fliC*	F<br>R	TATGCCGCTACATATGATGAG<br>TTAACGCAGTAAAGAGAG	495	[3]
4	*S.* Typhimurium	*Stm*	F<br>R	TTGTTCACTTTTTACCCCTGAA<br>CCCTGACAGCCGTTAGATATT	401	[4]
5	Genus *Salmonella*	*invA*	F<br>R	GTGAAATTATCGCCACGTTCGGGCAA<br>TCATCGCACCGTCAAAGGAACC	284	[5]

Table S2. Secondary metabolites in crude extract of *P. aeruginosa* (MC9) detected by ESI- MS/MS.

Serial no	Series	Structures of metabolites	Side Chains	Observed Peaks (*m/z*) [M+H]$^+$ *m/z*	[M+H]$^+$ Abundance **	[M-H]$^-$	MS/MS (Verified)*	MS/MS (Reported)	References
		**Pyocyanin**							
1		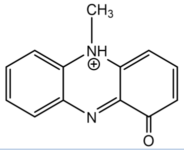					(+) 179, 183, 197,	(+) 179, 183, 197,	
		**(HAQs)**							
2	A	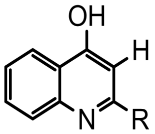	$C_{3:0}$–$\mathbf{C_3H_7}$	-	5	186	(-) 170, 158, 157, 144	-	
3			$C_{4:0}$–$\mathbf{C_4H_9}$	-	3	200	(-) 184, 172, 170, 158, 157, 144	-	
4			$C_{5:0}$–$\mathbf{C_5H_{11}}$	-	5	214	(-) 200, 186, 172, 170, 158, 157, 144	-	
5			$C_{7:1}$–$\mathbf{C_7H_{13}}$	242	3, 10	240	(+) 224, 214, 200, 186, 172, 160, 159, 146<br>(-) 222, 212, 198, 184, 170, 158, 157, 144	(+) 224, 213, 200, 194, 186, 185, 184, 172, 159, 146<br>(-) 198, 184, 172, 170, 158, 157, 144, 143	[6]
6			$C_{7:0}$–$\mathbf{C_7H_{15}}$	244	60, 40	242	(+) 226, 186, 172, 160, 159, 146<br>(-) 228, 214, 198, 184, 170, 158, 157, 144, 143	(+) 226, 186, 172, 159, 146<br>(-) 228, 198, 170, 158, 158, 144, 143.	[7]
7			$C_{8:1}$–$\mathbf{C_8H_{15}}$	256	2	-	(+) 238, 228, 214, 210, 200, 186, 172, 168, 160, 159, 146	-	[8]
8			$C_{8:0}$–$\mathbf{C_8H_{17}}$	258	9	-	(+) 240, 228, 214, 200, 198, 186, 172, 160, 159, 146	(+) 244, 230, 226, 216, 202,198, 188, 186, 184, 174, 172,170, 162, 160, 159, 156, 146,132.	[7]

9			$C_{9:1}$–$\mathbf{C_9H_{17}}$	270	24, 15	268	(+) 242, 228, 214, 200, 186, 172, 160, 159, 146<br>(-) 240, 212, 198, 184, 170, 158, 157, 144	(+) 228, 200, 186, 185, 184,173, 172, 160, 159.<br>(−) 226, 198, 184, 173, 170,158, 157, 144, 143.	[6]
10			$C_{9:0}$–$\mathbf{C_9H_{19}}$	272	34, 30	270	(+) 254, 242, 228, 214, 200, 186, 172, 160, 159, 146<br>(-) 242, 226, 212, 198, 184, 170, 158, 157, 144	(+) 258, 254, 240, 226, 216, 202, 198, 188, 186, 184, 172, 162, 160, 159, 146, 132<br>(-) 242, 226, 212, 198, 184, 170, 157	[7]
11			$C_{10:1}$–$\mathbf{C_{10}H_{19}}$	284	4	-	(+) 266, 256, 242, 228, 214, 200, 186, 172, 160, 159	-	
12			$C_{11:1}$–$\mathbf{C_{11}H_{21}}$	298	4, 25	296	(+) 280, 270, 256, 242, 228, 214, 200, 186, 172, 160, 159, 146<br>(-) 268, 254, 240, 226, 212, 198, 184, 170, 158, 157, 143	(+) 270, 256, 242, 228, 214, 200, 186, 172, 160, 159<br>(+) 160, 174	[6, 8]
13			$C_{13:2}$–$\mathbf{C_{13}H_{24}}$	-	30	322	(-) 294, 196, 184, 170, 158, 157	-	
14			$C_{13:1}$–$\mathbf{C_{13}H_{25}}$	-	10	324	(-) 306, 296. 282, 268, 254, 240, 226, 212, 198, 184, 170, 158, 157, 143	(-) 306, 296, 290, 286, 280, 260, 246, 244, 242, 223, 205, 184, 170, 158	[6]
15	B	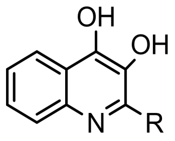	$C_{6:0}$–$\mathbf{C_6H_{13}}$	246	6	-	(+) 228, 218, 214, 200, 190, 186, 176, 174, 173, 172, 162, 160, 159	-	[9]
16			$C_{7:0}$–$\mathbf{C_7H_{15}}$	260	6, 50	258	(+) 242, 224, 214, 200, 186, 172, 162, 160, 159.<br>(-) 240, 230, 214, 186, 174, 173, 159, 144.	(+) 242, 186, 175, 172, 162, 159<br>(+) 188, 175<br>(-) 241, 240, 230, 214, 187, 173, 172, 159, 144	[6, 10]
17	C	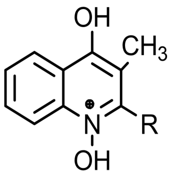	$C_{8:1}$–$\mathbf{C_8H_{15}}$	286	6	-	(+) 268, 258, 254, 244, 240, 230, 226, 216, 212, 202, 200, 188, 186, 174, 162, 160, 146	(+) 268, 258, 240, 226, 216, 212, 202, 198, 188, 186, 184, 174, 172, 162, 160, 159, 146, 132	[6]
18				-	10	286	(-) 268, 258, 242, 226, 214, 200, 186, 174, 173, 159, 144	(-) 269, 268, 258, 242, 186, 174, 170, 159, 158,	[6]

								157, 144	
19			$C_{8:0}$–**C_8H_{17}**	288	4	-	(+) 270, 260, 242, 228, 214, 200, 186, 162, 146	(+)272, 186, 172, 159, 146<br>(+) 188, 186, 172, 159, 144	[6, 8, 10]
20	D	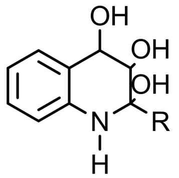	$C_{4:0}$–**C_4H_7**	-	5	236	(-) 200, 172, 158, 144	-	
21			$C_{7:0}$–**C_7H_{15}**	-	100	278	(-) 260, 250, 242, 222, 208, 194, 186, 170, 158, 157, 144	(-) 242, 170, 158, 157, 144	[6]
22			$C_{11:1}$–**$C_{11}H_{21}$**	-	15	332	(-) 296, 268, 254, 240, 170, 157, 144	-	
23	E	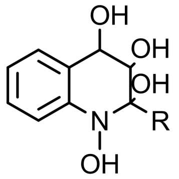	$C_{7:0}$–**C_7H_{15}**	-	90	294	(-) 258, 196, 194, 184, 170, 158, 157, 144	(-) 279, 266, 258, 249, 248, 236, 223, 208, 196, 194, 184, 183, 170, 158	[6]
24			$C_{8:1}$–**C_8H_{15}**	-	88	306	(-) 278, 270, 262, 248, 236, 224, 174, 158, 144	(-) 306, 270, 252, 236, 226, 198, 183, 170, 158, 157, 144	[6]
		**Rhamnolipids**							
	**Mono-rhamno-di-lipidic congers**								
25	G	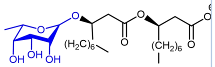	**Rha-C_{10}-C_{10}**	-	45	503	(-) 339, 333, 169.	(-) 474, 362, 339, 334, 324, 306, 169	[7]
	**Di-rhamno-di-lipidic congers**								
26	H	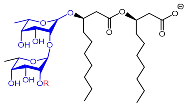	**Rha-Rha-C_{10}-C_{10}**	-	7	649	(-) 479, 339, 333, 169.	(-) 479, 339	[7]
27			**Decenoyl-Rha-Rha-C_{10}-C_{12}**	-	6	677	(-) 479, 333, 169.	-	[7]
28			**Decenoyl-Rha-Rha-C_{14}-C_{14}**		3	761	(-) 703, 649, 475, 451, 339,	-	[7]

*MS/MS verified results are 2% normalized (Only those fragments (m/z) are mentioned whose abundance is >2%).

**Percent relative abundance of peaks with respect to the base peak at *m/z* 278 (derived from negative ionization mode).

Table S3: Phenotypic antimicrobial resistance profiles of clinical isolates used in minimum inhibitory concentration determination and synergism assays.

Sr. No.	Antimicrobials	Code-Disc potency (μg)	*S.* Typhimurium W20	*S.* Typhi 29C	*E. coli.* SS1
1	Sulfamethoxazole /Trimethoprim	SXT-23.75/1.25	R	R	R
2	Nalidixic acid	NA-30	R	R	R
3	Ampicillin	AMP-10	S	S	R
4	Chloramphenicol	C-30	S	S	R
5	Aztreonam	ATM-30	IR	IR	IR
6	Amoxicillin/ Clavulanic acid	AMC-30	IR	S	IR
7	Gentamicin	GEN-10	S	R	R
8	Ceftriaxone	CRO-30	IR	IR	R
9	Ciprofloxacin	CIP-5	R	S	R

R: Resistant; IR: Intermediate resistant; S: Sensitive.

Table S4: Pyocyanin coactivity with antimicrobials against Enterobacteriaceae MDR clinical isolates.

Sr. No.	Antimicrobial	Code (µg/disc)	Increase in fold area of growth inhibiting area demonstrating combination effects with pyocyanin (%)								
			S. Typhimurium-W20			*S.* Typhi-29S			*E. coli*-SS1		
			10 µg/disc	25 µg/disc	30 µg/disc	10 µg/disc	25 µg/disc	50 µg/disc	10 µg/disc	25 µg/disc	50 µg/disc
1	Sulfamethoxazole / Trimethoprim	SXT 23.75/1.25	0 ± 0	300 ± 0*	0 ± 0	0 ± 0	0 ± 0	0 ± 0	0 ± 0	0 ± 0**	116.8 ± 6.6**
2	Nalidixic acid	NA 30	0 ± 0	0 ± 0	0 ± 0	0 ± 0	0 ± 0	0 ± 0	0 ± 0	0 ± 0	133.5 ± 6.9**
3	Ampicillin	AMP 10	(-) 15 ± 2.2	(-) 32.2 ± 1.8**	(-) 34.4 ± 1.8**	(-) 15.3 ± 2.3*	(-) 24 ± 2.0**	(-) 15.3 ± 2.2**	0 ± 0	0 ± 0	125 ± 0**
4	Chloramphenicol	C 30	(-) 31 ± 1.5	(-) 17 ± 0.4	(-) 18 ± 4.2	(-) 35 ± 0.3**	(-) 27.2 ± 3.7*	0 ± 0	0 ± 0	0 ± 0	226.1 ± 8.1**
5	Aztreonam	ATM 30	16.3 ± 1.7	16.3 ± 1.71	0 ± 0	11.4 ± 0	0 ± 0	23.4 ± 0	(-) 14 ± 2.6	(-) 21.5 ± 4.8*	(-) 31.66 ± 0.4**
6	Amoxicillin clavulanic acid	AMC 30	(-) 29 ± 2.0*	(-) 25.4 ± 0.8	0 ± 6.3	(-) 27 ± 0**	0 ± 0**	0 ± 0	0 ± 0**	0 ± 0**	0 ± 0**
7	Gentamicin	GEN 10	21 ± 0.6	25.9 ± 6.6*	9.7 ± 6.6	199.9 ± 13.1**	201.4 ± 11.9**	7 ± 5.7	(-) 40 ± 4.5	(-) 34.6 ± 4.5	(-) 29 ± 0
8	Ceftriaxone	CRO 30	32.9 ± 3.9**	0 ± 0	0 ± 0	0 ± 0**	0 ± 0	0 ± 0*	0 ± 0	0 ± 0	0 ± 0
9	Ciprofloxacin	CIP 5	40.3 ± 3.5	0 ± 0	0 ± 0	18.3 ± 2.4**	18.3 ± 2.4**	10.3 ± 2.2*	(-) 67 ± 4.6**	(-) 67 ± 4.6**	(-) 39 ± 0.2

Data are reported as the mean and SEM from three independent experiments (***P<0.001, **P< 0.01, *<0.05). Synergism **(+)** indicates that the combined treatment produced significantly greater growth inhibition than either agent alone. Antagonism **(−)** denotes reduced growth inhibition /enhanced bacterial growth in the combination compared with individual treatments. Indifferent effects indicate that the combined treatment did not produce a meaningful difference in growth inhibition relative to the single agent.

Table S5: Rhamnolipids coactivity with antimicrobials against Enterobacteriaceae MDR clinical isolates

Sr. No.	Antimicrobial	Code (µg/disc)	Increase in fold area of growth inhibiting area demonstrating combination effect with rhamnolipids (%)								
			S. Typhimurium-W20			*S.* Typhi-29S			*E. coli*-SS1		
			30 µg/disc	100 µg/disc	500 µg/disc	30 µg/disc	100 µg/disc	500 µg/disc	30 µg/disc	100 µg/disc	500 µg/disc
1	Sulfamethoxazole /Trimethoprim	SXT 23.75/1.25	0 ± 0	0 ± 0	0 ± 0	0 ± 0	0 ± 0	0 ± 0	0 ± 0	0 ± 0	0 ± 0
2	Nalidixic acid	NA 30	0 ± 0	0 ± 0	0 ± 0	0 ± 0	0 ± 0	36.1 ± 0	0 ± 0	0 ± 0	0 ± 0
3	Ampicillin	AMP 10	(-) 15.3 ± 2.2**	11.5 ± 1.3	0 ± 0	0 ± 0	6.42 ± 1.4	4.6 ± 0.08	0 ± 0	0 ± 0	36.1 ± 0**
4	Chloramphenicol	C 30	(-) 55.5 ± 4.1*	(-)61.6 ± 0**	(-)67.3 ± 0	0 ± 0	0 ± 0	0 ± 0	0 ± 0	0 ± 0	77.7 ± 0**
5	Aztreonam	ATM 30	(-) 37.0 ± 1.9**	(-) 45.7 ± 1.7**	(-) 53.7 ± 1.4**	13.7 ± 1.9	0 ± 0	0 ± 0	0 ± 0	0 ± 0	0 ± 0
6	Amoxicillin clavulanic acid	AMC 30	0 ± 0	0 ± 0	0 ± 0	0 ± 0	0 ± 0	0 ± 0	20.2 ± 1.3	0 ± 0	0 ± 0
7	Gentamicin	GEN 10	(-) 55.5 ± 4.1	(-) 55.5 ± 4.1	(-) 55.5 ± 4.1	0 ± 0	0 ± 0	0 ± 0**	0 ± 0	0 ± 0	0 ± 0
8	Ceftriaxone	CRO 30	34.0 ± 0**	10.8 ± 0**	22.1 ± 0**	0 ± 0	0 ± 0	0 ± 0	106.6 ± 0**	106.6 ± 0**	106.6 ± 0**
9	Ciprofloxacin	CIP 5	39.6 ± 0**	61.9 ± 0**	0 ± 0	0 ± 0	0 ± 0	0 ± 0	0 ± 0	0 ± 0	0 ± 0

Data are reported as the mean and SEM from three independent experiments (***P<0.001, **P< 0.01, *<0.05). Synergism **(+)** indicates that the combined treatment produced significantly greater growth inhibition than either agent alone. Antagonism **(−)** denotes reduced growth inhibition /enhanced bacterial growth in the combination compared with individual treatments. Indifferent effects indicate that the combined treatment did not produce a meaningful difference in growth inhibition relative to the single agent.

Table S6: HHQ coactivity with antimicrobials against Enterobacteriaceae MDR clinical isolates

Sr No.	Antimicrobial	Code (µg/disc)	Increase in fold area of growth-inhibiting area demonstrating combination effects with HHQ (%)								
			S. Typhimurium-W20			*S.* Typhi-29S			*E. coli*-SS1		
			10 µg/disc	50 µg/disc	100 µg/disc	10 µg/disc	50 µg/disc	100 µg/disc	10 µg/disc	50 µg/disc	100 µg/disc
1	Sulfamethoxazole /Trimethoprim	SXT 23.75/1.25	0 ± 0	0 ± 0	0 ± 0	0 ± 0	0 ± 0	0 ± 0	0 ± 0	0 ± 0	0 ± 0
2	Nalidixic acid	NA 30	0 ± 0	0 ± 0	0 ± 0	0 ± 0	0 ± 0	0 ± 0	0 ± 0	0 ± 0	0 ± 0
3	Ampicillin	AMP 10	0 ± 0	0 ± 0	0 ± 0	0 ± 0	0 ± 0	0 ± 0	37.9 ± 18	0 ± 0	257.4 ± 17.3**
4	Chloramphenicol	C 30	(-) 73.3 ± 0.6**	(-) 73.3 ± 0.6**	(-) 73.3 ± 0.6**	36.1 ± 0**	56.2 ± 0**	26.5 ± 0**	0 ± 0	0 ± 0	77.7 ± 0**
5	Aztreonam	ATM 30	11.8 ± 0.1	11.8 ± 0.1	0 ± 0	11.4 ± 0	11.4 ± 0	11.4 ± 0	12.1 ± 0	12.1 ± 0	12.1 ± 0
6	Amoxicillin clavulanic acid	AMC 30	14.7 ± 1.0**	0 ± 0	14.7 ± 1.0**	44.0 ± 1.0**	44.0 ± 0**	10.2 ± 0	141.9 ± 1.0	136.3 ± 4.6	141.9 ± 0
7	Gentamicin	GEN 10	0 ± 0	0 ± 0	0 ± 0	0 ± 0	0 ± 0	0 ± 0	0 ± 0	0 ± 0	30.6 ± 0**
8	Ceftriaxone	CRO 30	28.0 ± 2.0**	28.0 ± 2.0**	10.8 ± 0**	23.4 ± 0**	49.3.0 ± 0*	23.4 ± 0**	0 ± 0	89.0 ± 0	11.9 ± 0
9	Ciprofloxacin	CIP 5	85.9 ± 0**	39.6 ± 0**	39.6 ± 0**	0 ± 0	0 ± 0	0 ± 0	0 ± 0	0 ± 0	0 ± 0

Data are reported as the mean and SEM from three independent experiments (***P<0.001, **P< 0.01, *<0.05). Synergism **(+)** indicates that the combined treatment produced significantly greater growth inhibition than either agent alone. Antagonism **(−)** denotes reduced growth inhibition /enhanced bacterial growth in the combination compared with individual treatments. Indifferent effects indicate that the combined treatment did not produce a meaningful difference in growth inhibition relative to the single agent.

Table S7: PQS coactivity with antimicrobials against Enterobacteriace*a*e clinical MDR isolates

Sr No.	Antimicrobial	Code (µg/disc)	Increase in fold area of growth inhibiting area demonstrating combination effects with PQS (%)								
			S. Typhimurium-W20			*S.* Typhi-29C			*E. coli*-SS1		
			10 µg/disc	50 µg/disc	100 µg/disc	10 µg/disc	50 µg/disc	100 µg/disc	10 µg/disc	50 µg/disc	100 µg/disc
1	Sulfamethoxazole /Trimethoprim	SXT 23.75/1.25	0 ± 0	0 ± 0**	0 ± 0	0 ± 0	0 ± 0	0 ± 0	0 ± 0	0 ± 0	0 ± 0
2	Nalidixic acid	NA 30	0 ± 0	0 ± 0	0 ± 0	0 ± 0	0 ± 0	0 ± 0	0 ± 0	0 ± 0	0 ± 0
3	Ampicillin	AMP 10	31.7 ± 0.4**	14 ± 5.7	14 ± 5.7	(-) 31.8 ± 2.1**	(-) 34.4 ± 0**	(-) 34.4 ± 0*	0 ± 0	0 ± 0	0 ± 0
4	Chloramphenicol	C 30	0 ± 0	0 ± 0	0 ± 0	26.5 ± 0**	8.5 ± 0*	8.5 ± 0*	0 ± 0	0 ± 0	0 ± 0
5	Aztreonam	ATM 30	23.9 ± 0.7	23.9 ± 0.7	23.9 ± 0.7	35.4 ± 0.5*	35.4 ± 0.5**	11.21 ± 0.1	0 ± 0	0 ± 0	0 ± 0
6	Amoxicillin clavulanic acid	AMC 30	30.6 ± 0**	47.4 ± 0**	14.7 ± 1.0*	0 ± 0	0 ± 0	0 ± 0	108.6 ± 0***	108.6 ± 0***	108.6 ± 0***
7	Gentamicin	GEN 10	(-)45.7 ± 0*	(-)45.7 ± 0*	(-)45.7 ± 0*	(-) 60.4 ± 3.9**	(-) 60.4 ± 3.9**	(-) 60.4 ± 3.9**	0 ± 0	0 ± 0	0 ± 0
8	Ceftriaxone	CRO 30	22 ± 0**	22.1 ± 0**	22.1 ± 0**	(-) 30.5 ± 0	(-) 30.5 ± 0	(-) 30.5 ± 0	0 ± 0	0 ± 0**	0 ± 0
9	Ciprofloxacin	CIP 5	39.6 ± 0*	85.9 ± 0**	39.6 ± 0*	45.8 ± 2.8*	93.4 ± 3.7**	73.6 ± 3.3**	0 ± 0**	0 ± 0	0 ± 0

Data are reported as the mean and SEM from three independent experiments (***P<0.001, **P< 0.01, *<0.05). Synergism **(+)** indicates that the combined treatment produced significantly greater growth inhibition than either agent alone. Antagonism **(−)** denotes reduced growth inhibition /enhanced bacterial growth in the combination compared with individual treatments. Indifferent effects indicate that the combined treatment did not produce a meaningful difference in growth inhibition relative to the single agent.

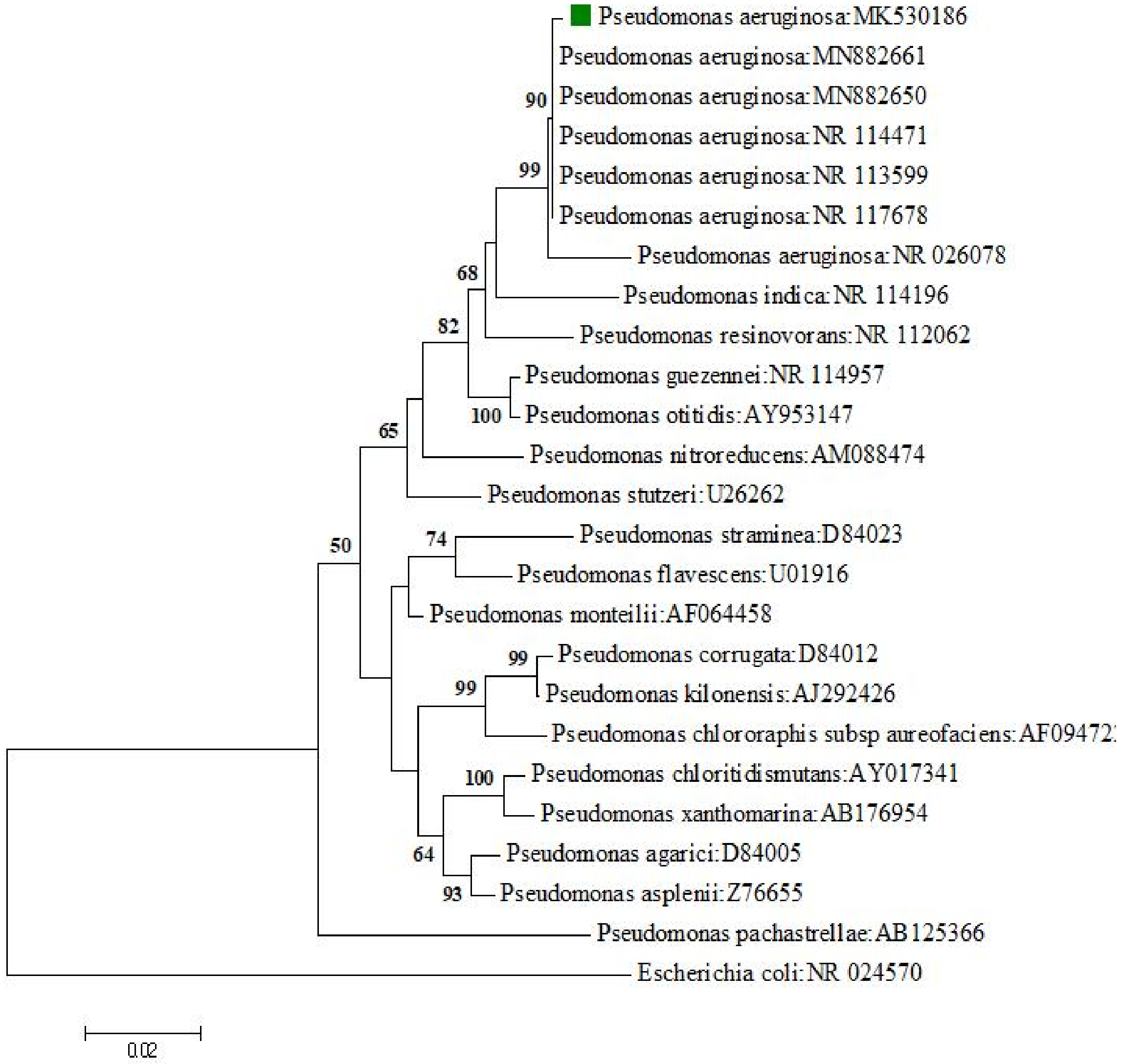

Figure S1. Phylogenetic tree showing *P. aeruginosa* isolates compared with several other representatives of Pseudomonadaceae family. The dendrogram was reconstructed using the Maximum Likelihood (ML) method and Mega 7.0 software based on TN+G model with concatenated 16S rRNA sequences. Bootstraps values were higher than 50%, as indicated with numbers on the branch nodes, were calculated from 1000 replicates. Characters after the species names are GenBank identifiers for the respective 16S rRNA sequences. The green square marks isolate *P. aeruginosa* MC9, which was used for this study.

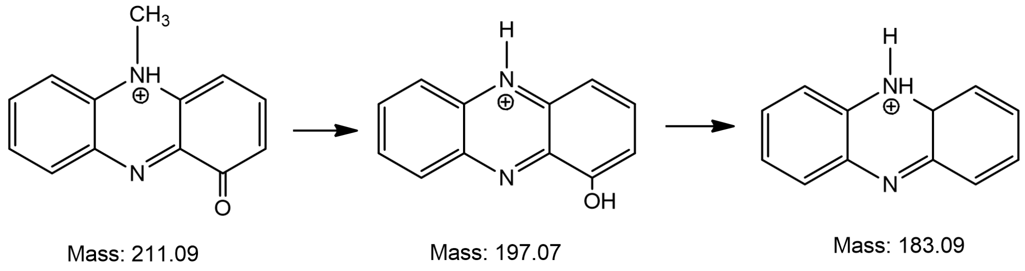

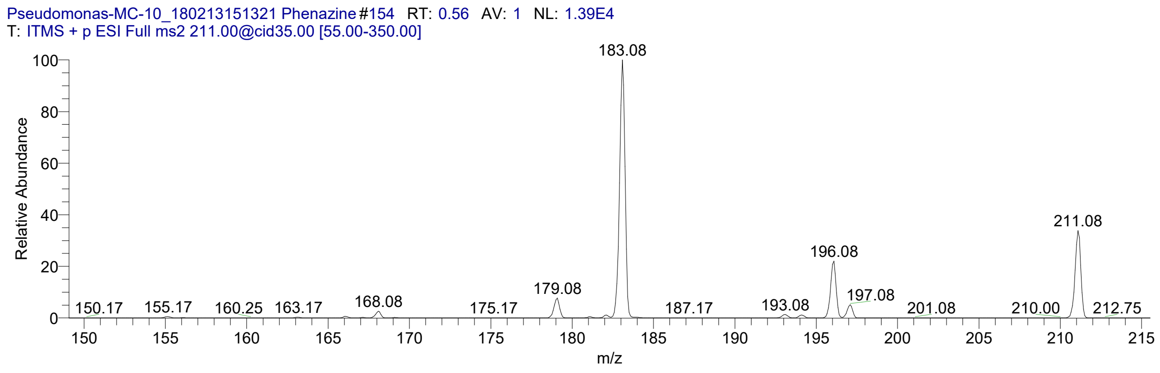

Figure S2. Putative structures of daughter ions produced by fragmentation of the molecular ion at *m/z* 211 $[M+H]^+$.

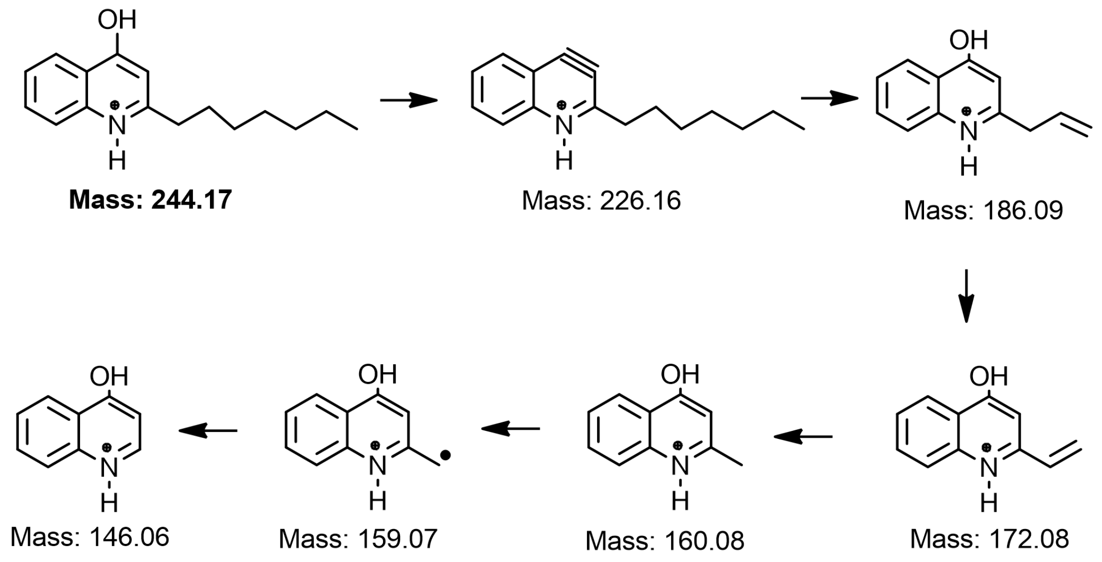

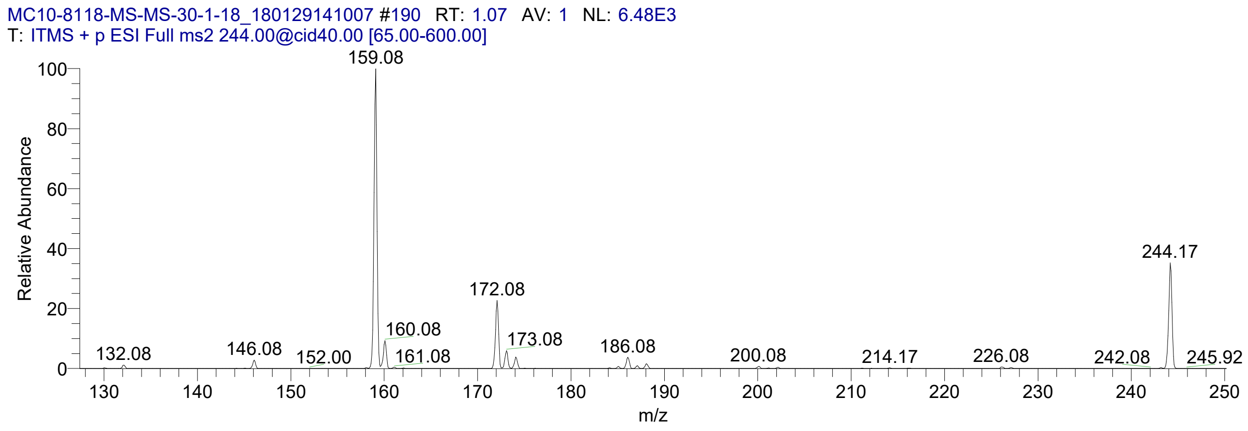

Figure S3. Putative structures of daughter ions produced by fragmentation of the molecular ion at *m/z* 244 $[M+H]^+$

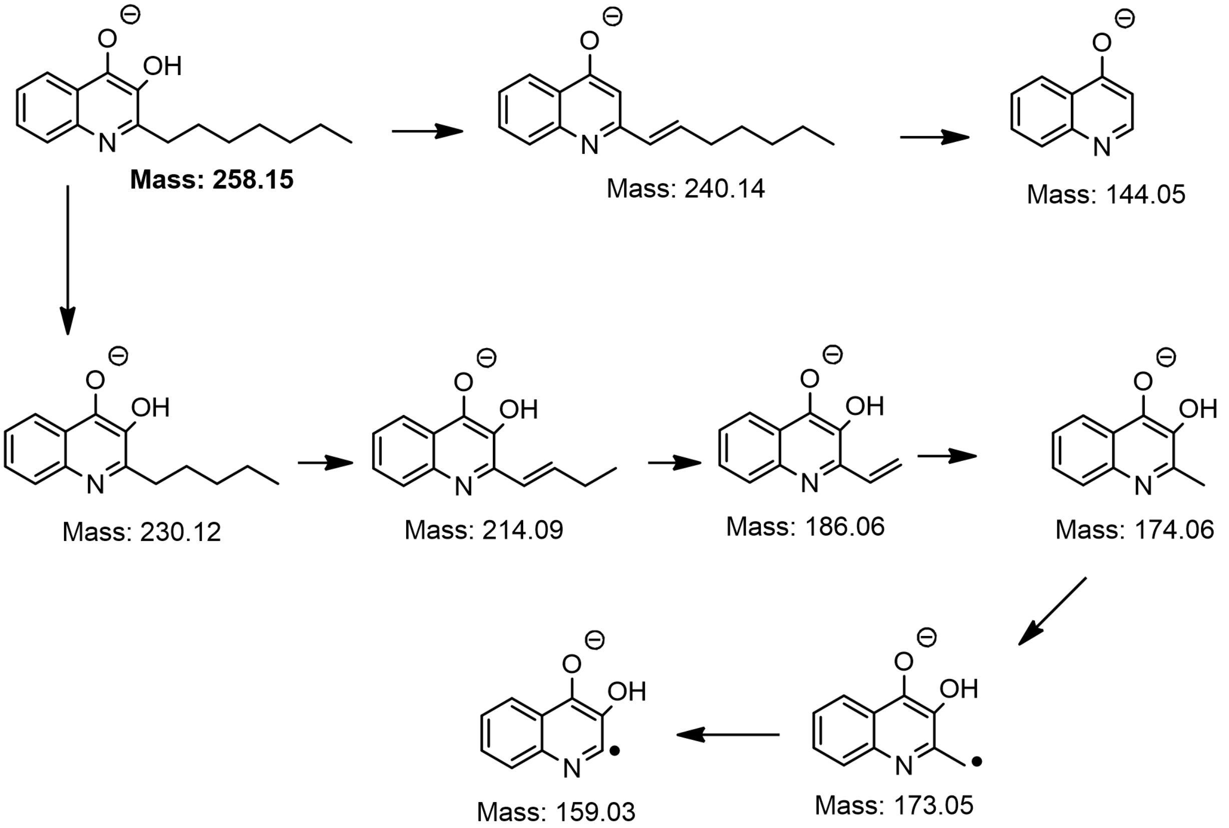

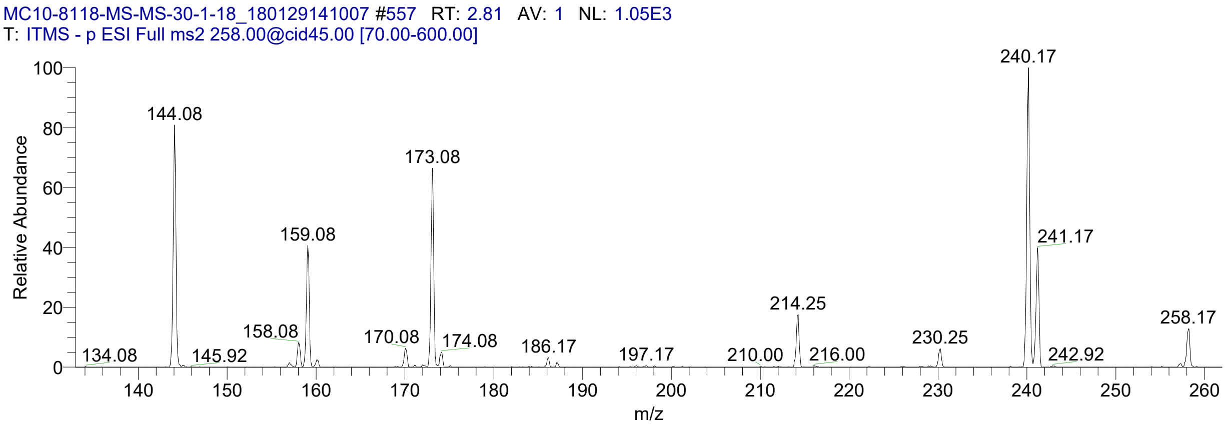

Figure S4. Putative structures of daughter ions produced by fragmentation of the molecular ion at *m/z* 258 [M-H]⁻

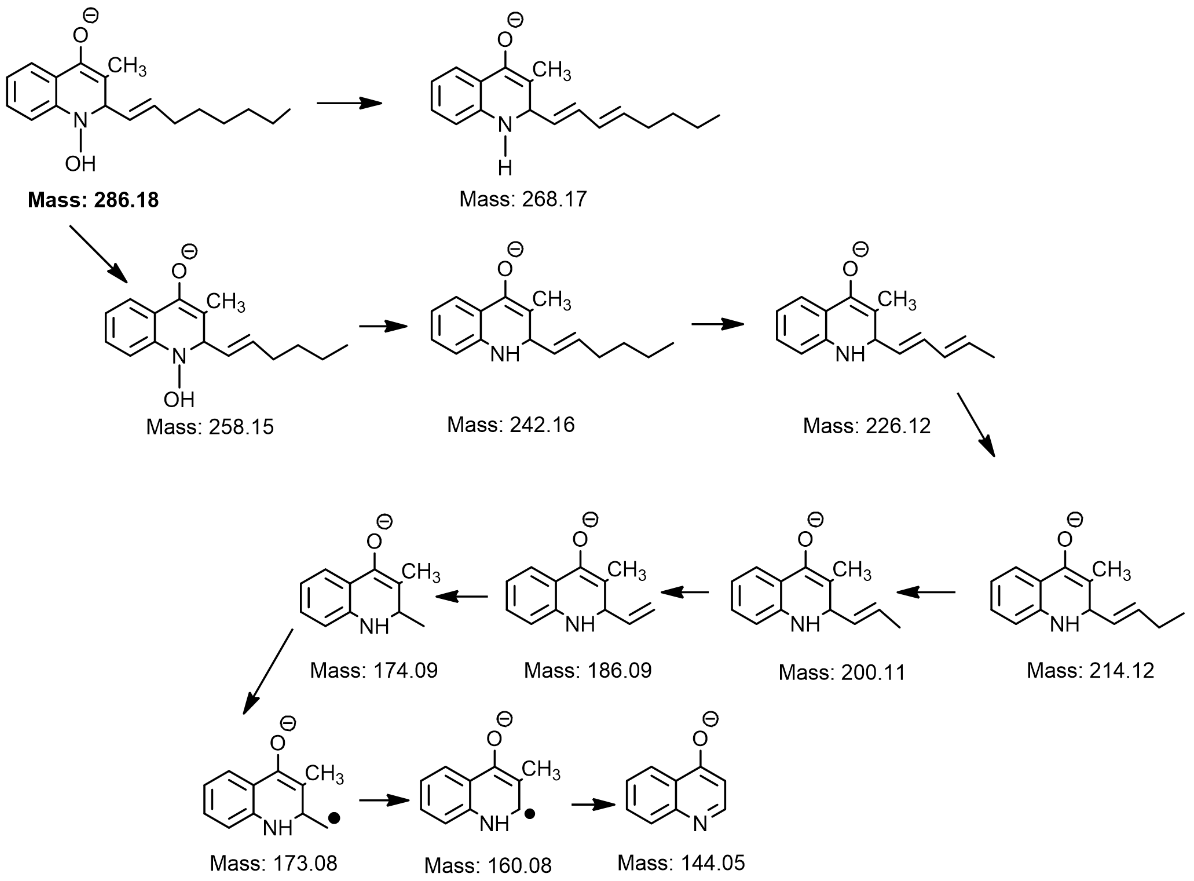

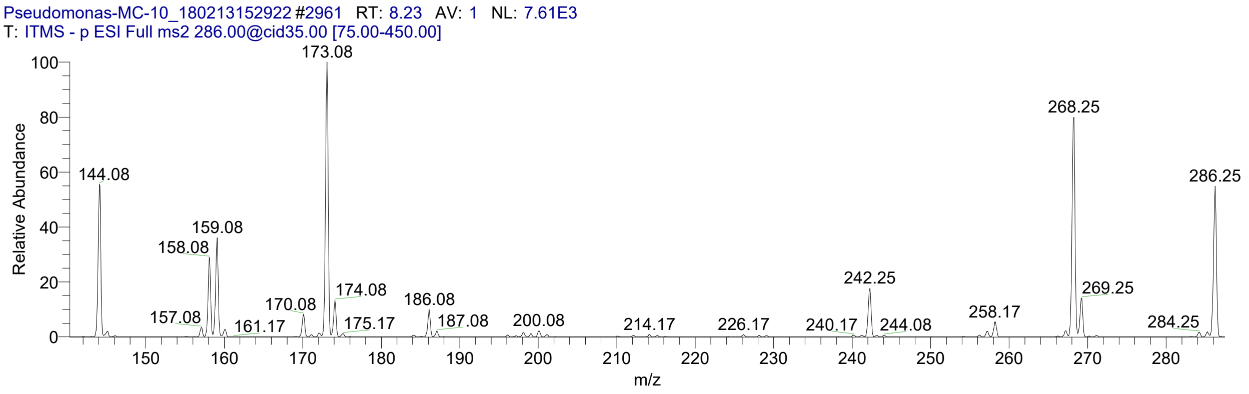

Figure S5: Putative structures of the fragment ion produced by CID (energy 35.0) of the ion at *m/z* 286 [M-H]$^-$

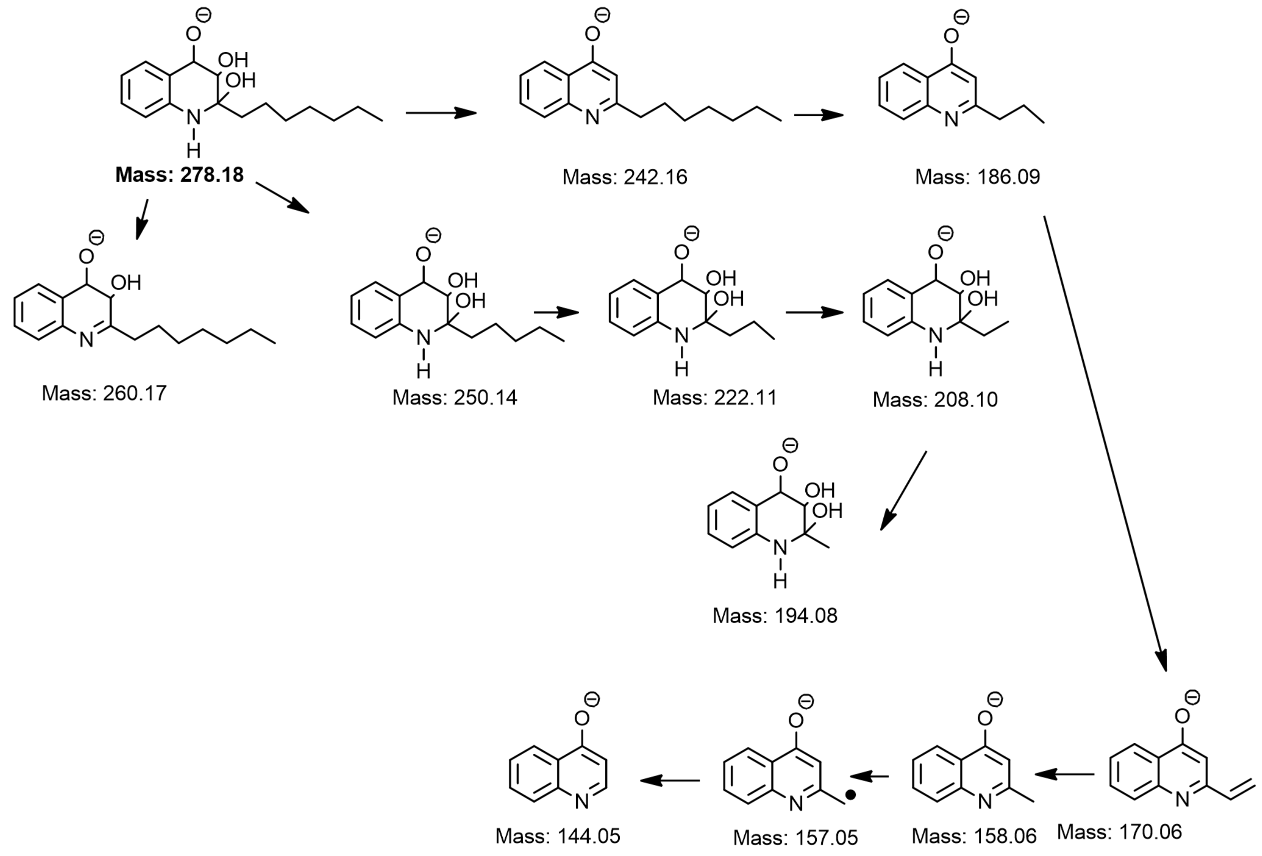

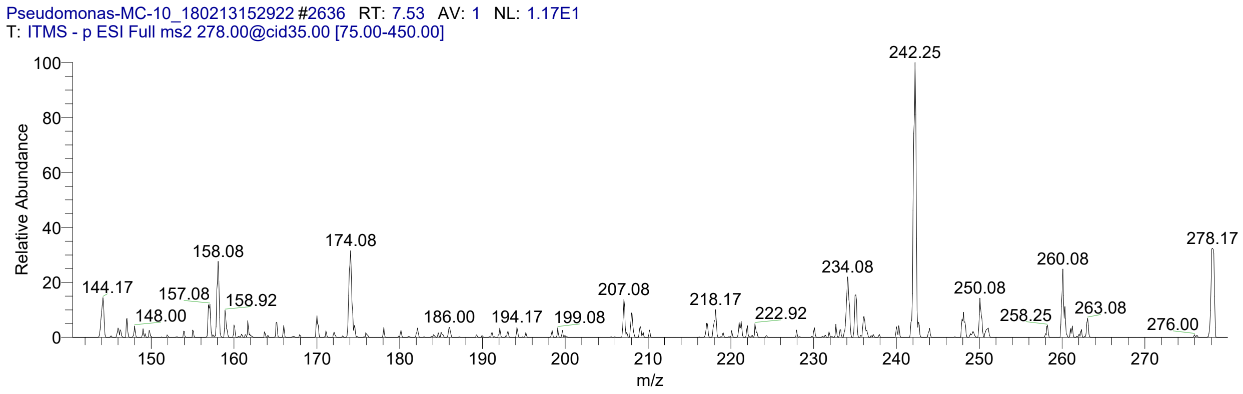

Figure S6. Putative structures of the daughter ions produced by fragmentation of the ion at *m/z* 278 $[M\text{-}H]^-$

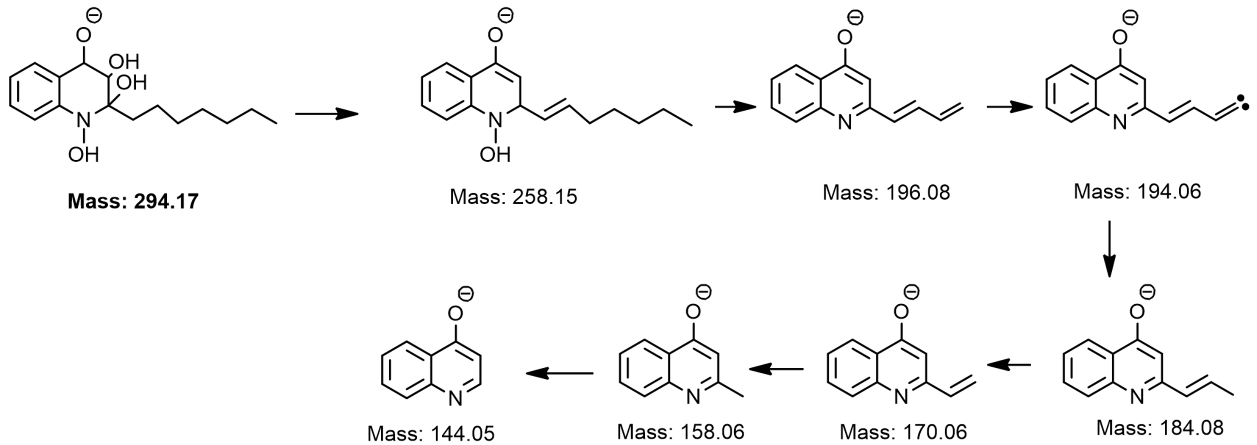

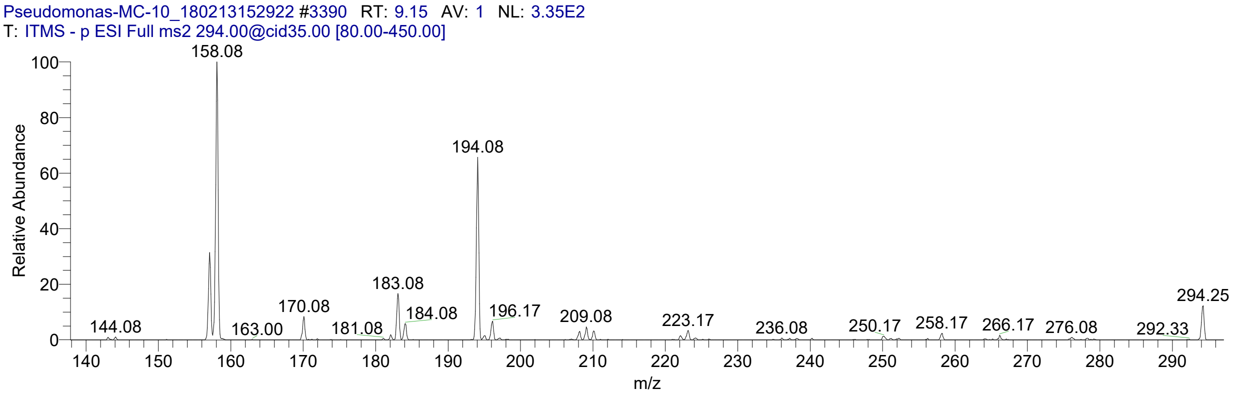

Figure S7. Profiling of the fragmentation data generated through the tandem mass spectrometry of the ion at *m/z* 294 using CID (energy 35.0) at [M-H] $^-$

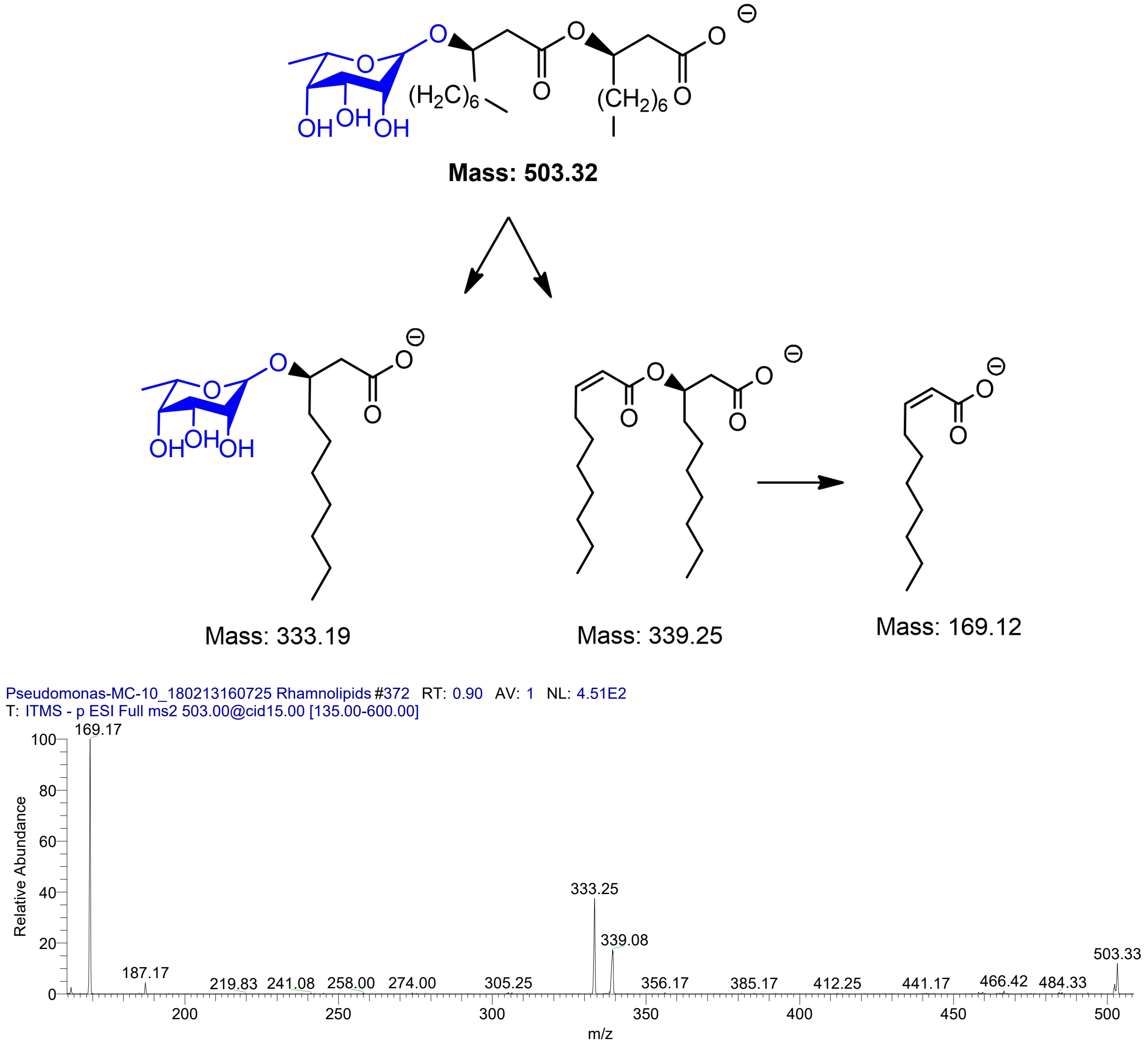

Figure S8. Profiling of the fragmentation data generated through the tandem mass spectrometry of the ion at *m/z* 503 [M-H] $^-$ (Mono-rhamno-di-lipidic congers) using CID (energy 15.0) full scan at negative ion mode.

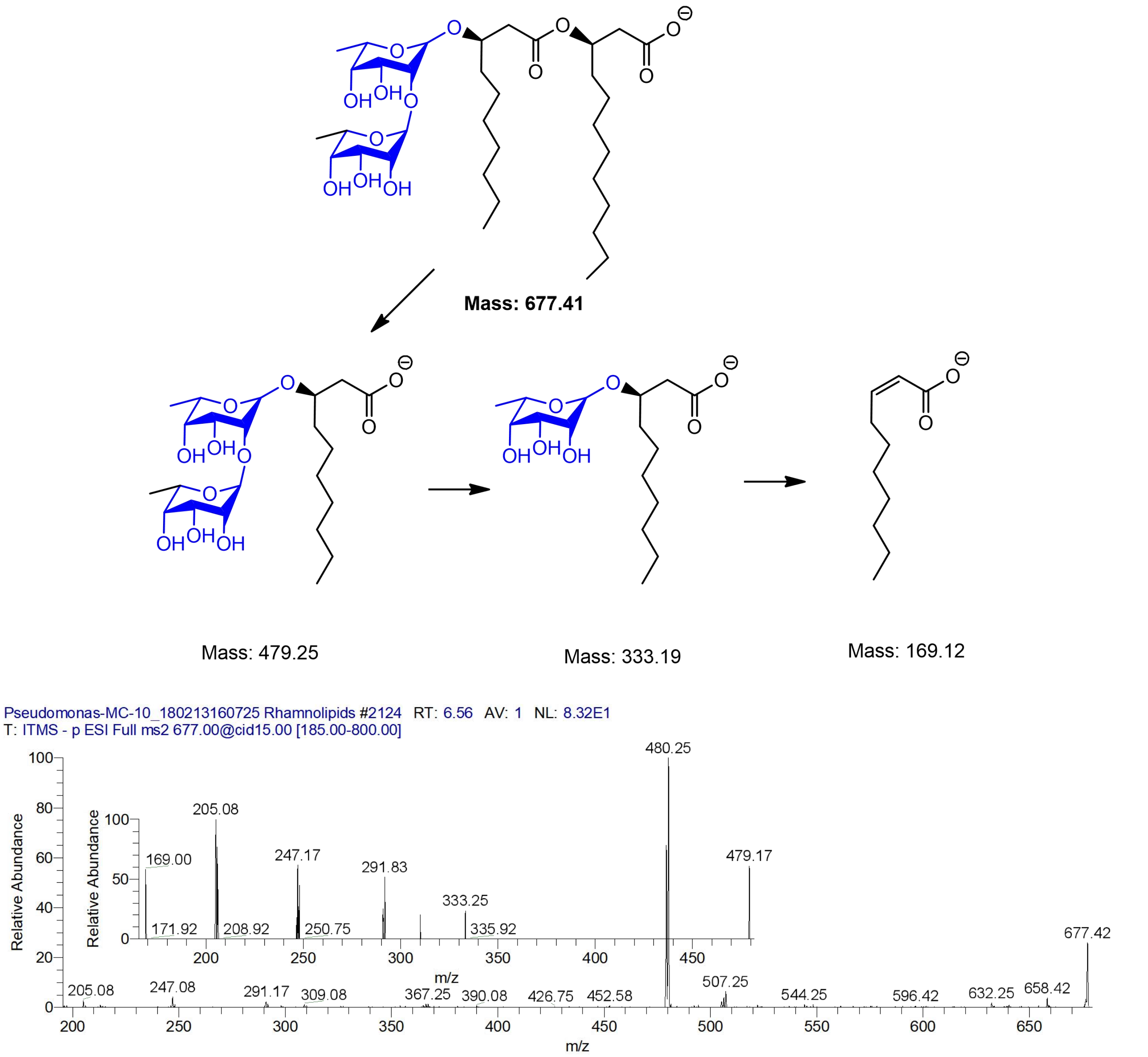

Figure S9. Profiling of the fragmentation data generated through the tandem mass spectrometry of the ion at *m/z* 677 [M-H]$^-$. (Di-rhamno-di-lipidic congers) using CID (energy 15.0) full scan at negative ion mode.

References:

1. Awan, A.B., et al., *Detection of synergistic antimicrobial resistance mechanisms in clinical isolates of Pseudomonas aeruginosa from post-operative wound infections.* Applied Microbiology and Biotechnology, 2021. **105**: p. 9321-9332.
2. Heininger, A., et al., *PCR and blood culture for detection of Escherichia coli bacteremia in rats.* Journal of clinical microbiology, 1999. **37**(8): p. 2479-2482.
3. Song, J.-H., et al., *Detection of Salmonella typhi in the blood of patients with typhoid fever by polymerase chain reaction.* Journal of Clinical Microbiology, 1993. **31**(6): p. 1439-1443.
4. Liora, M., et al., *Antimicrobial Resistance Evaluation of Pathogen Salmonella Strains Isolated in Pork and Poultry Meat.* Bulletin of the University of Agricultural Sciences & Veterinary Medicine Cluj-Napoca. Veterinary Medicine, 2013. **70**(2).
5. Moussa, I., et al., *Using molecular techniques for rapid detection of Salmonella serovars in frozen chicken and chicken products collected from Riyadh, Saudi Arabia.* African Journal of Biotechnology, 2010. **9**(5).
6. Yasmin, S., et al., *Biocontrol of bacterial leaf blight of rice and profiling of secondary metabolites produced by rhizospheric Pseudomonas aeruginosa BRp3.* Frontiers in Microbiology, 2017. **8**: p. 1895.
7. Akbar, N., et al., *Gut bacteria of Cuora amboinensis (turtle) produce broad-spectrum antibacterial molecules.* Scientific reports, 2019. **9**(1): p. 1-19.
8. Vial, L., et al., *Burkholderia pseudomallei, B. thailandensis, and B. ambifaria produce 4-hydroxy-2-alkylquinoline analogues with a methyl group at the 3 position that is required for quorum-sensing regulation.* Journal of bacteriology, 2008. **190**(15): p. 5339-5352.
9. Déziel, E., et al., *Analysis of Pseudomonas aeruginosa 4-hydroxy-2-alkylquinolines (HAQs) reveals a role for 4-hydroxy-2-heptylquinoline in cell-to-cell communication.* Proceedings of the National Academy of Sciences, 2004. **101**(5): p. 1339-1344.
10. Lépine, F., et al., *Electrospray/mass spectrometric identification and analysis of 4-hydroxy-2-alkylquinolines (HAQs) produced by Pseudomonas aeruginosa.* Journal of the American Society for Mass Spectrometry, 2004. **15**(6): p. 862-869.
